# The balloon model revisited

**DOI:** 10.21203/rs.3.rs-11197888/v1

**Published:** 2026-09-30

**Authors:** John C. Gore, Tzu-Wei Li, Yang Jiao, Abbe L. Knutsson, Warren W. Boschen, Muwei Li, Kurt Schilling, Adam W. Anderson, Zhaohua Ding, Yurui Gao

**Affiliations:** 1 Vanderbilt University Institute of Imaging Science, Vanderbilt University, Nashville, TN 37232, USA; 2 Department of Biomedical Engineering, Vanderbilt University, Nashville, TN 37232, USA; 3 Department of Radiology and Radiological Sciences, Vanderbilt University Medical Center, Nashville, TN 37232, USA; 4 Materials Science and Engineering, Arizona State University; Tempe, 85287, USA; 5 Department of Electrical and Computer Engineering, Vanderbilt University, Nashville, TN 37232, USA

**Keywords:** Hemodynamic response function, BOLD, fMRI, balloon model

## Abstract

Functional MRI based on the blood oxygenation level–dependent (BOLD) effect infers neural activity from hemodynamic changes through a hemodynamic response function (HRF). We revisit the balloon model of the BOLD effect and show that Grubb’s empirical flow–volume power law can be used to determine how vascular transit time varies with flow and is governed by a single index of venous distensibility. Linearizing the resulting equations yields an explicit closed-form HRF, expressed in physiologically recognizable parameters, that reproduces the canonical response. The initial dip, time to peak and poststimulus undershoot are each traced to identifiable terms, and the relations assumed in calibrated BOLD signals emerge as outputs. When oxygen consumption is specified independently of flow, as in earlier generalizations, the analytic solution quantifies the specific relationships between the latency and depth of an initial dip and a neurovascular delay. The new model will prove useful for explaining variations in HRF in white matter, across the brain, and between different populations.

## Introduction

We reconsider the model and equations of Buxton et al. (1), who described the blood oxygenation-level dependent (BOLD) signal (2), which is the basis for functional MRI of the brain and spine. FMRI has become well established as the premier noninvasive imaging technology for mapping the functional organization of the brain. FMRI acquisitions are used to detect localized activations caused by changes in a stimulus condition, to detect transient signals evoked by very short stimuli (event-related fMRI) and to detect fluctuations in the baseline signals in trains of images obtained while subjects are in a “resting state” (3). Correlations between such fluctuations are deemed to depict functional connectivity within and between networks.

Although data-driven methods for analyzing sets of fMRI images, such as independent component analysis (ICA), have been used in various applications, the most common approach for deriving measures of brain activity involves the use of a general linear model that assumes a specific relationship between a transient change in neural activity and the BOLD response, called the hemodynamic response function (HRF) (4, 5). There is a growing body of literature on the appropriate design and selection of HRF for optimizing sensitivity to brain activity (6, 7), although rather less emphasis has been placed on mechanistic models that predict the form of HRF. Analytical treatments for hemodynamic models have been proposed previously. Friston et al. (4) placed the balloon model in state-space form and derived Volterra kernels, the first-order kernel being an impulse-response representation of the HRF; Robinson, Aquino, Pang and colleagues linearized spatially extended, poroelastic models of cortical hemodynamics, formed their transfer functions, identified the poles and inverted them to obtain closed-form responses and response modes (8–12). The present work follows a similar route (linearize, transform and invert) for a lumped model, with the advantage that the resulting expressions are compact and that each term corresponds to an identifiable physiological quantity.

BOLD signals from neural tissues are indirectly related to underlying neural metabolic activity (13, 14), and the nature of that coupling profoundly affects the temporal response of the BOLD signal to transient changes in conditions. Understanding the factors that govern HRF is key to interpreting the variations in HRF that are evident across the brain, between individuals, and, in particular, between gray and white matter (15–17). Moreover, previous investigators have attempted to calibrate the BOLD effect in terms of underlying metabolic and physiological properties (18–20) and have relied on concepts derived from the balloon model and empirical observations for validation. Here, we revisit the assumptions of previous models and rewrite their constitutive equations to derive a more complete theoretical explanation of the BOLD effect and HRF and quantify how specific features are related to underlying, measurable physiological parameters. We first describe the original model of Buxton et al. (1) as modified by Obata (21) and identify the conditions under which the BOLD signal is linearly proportional to the deoxyhemoglobin content. We then recast Grubb’s empirical flow–volume power law (22) in terms of vascular transit times and the mechanical properties of the draining vessels. Using these new insights, we explain how the exact shape of the HRF is governed by specific factors. Following earlier versions of the model that treat oxygen consumption as an input independent of flow (21, 23), we then remove the assumption that oxygen consumption is determined by flow, which permits increases in metabolism to occur before changes in flow and cause an initial dip, and derive closed-form expressions for that dip. We finally solve the linearized system exactly, first from oxygen consumption to signal and then for the complete response to a neural event. Numerical results are then presented, including predictions of the HRF for different underlying parameters and fits of the theoretical functions to the canonical HRF used in standard analysis software. These fits are intended as a practical demonstration that the model reproduces the standard response with physiologically plausible parameters.

The symbols used are defined at first use and are listed in Table S1.

## Results

### (a) The balloon model of Buxton et al. was modified from Obata

The original balloon model (1) treats the postcapillary venous compartment in tissue as an expandable balloon, fed by an inflow $F_{\text{in}}(t)$ and drained by an outflow $F_{\text{out}}(t)$. Two time-varying quantities of relevance are the venous blood volume V(t) and the total quantity of deoxyhemoglobin it contains Q(t). Conservation of blood volume and deoxyhemoglobin levels

(1) $$\frac{dV}{dt}=F_{\text{in}}(t)-F_{\text{out}}(t)$$

(2) $$\frac{dQ}{dt}=F_{\text{in}}(t)C_{a}E(t)-F_{\text{out}}(t)\frac{Q}{V}$$

where $C_{a}$ is the arterial oxygen content, E(t) is the net oxygen extraction fraction, and Q/V is the venous deoxyhemoglobin concentration. It is convenient to normalize each quantity by its resting value, written as $v=V/V_{0},q=Q/Q_{0},f_{\text{in}}=F_{\text{in}}/F_{0}$ and $f_{\text{out}}=F_{\text{out}}/F_{0}$, all of which are unity at rest.

The mean transit time of blood through the compartment is $\tau_{0}$

(3) $$\tau_{0}=\frac{V_{0}}{F_{0}}$$

Equations (1) and (2) become

(4) $$\tau_{0}\frac{dv}{dt}=f_{\text{in}}(t)-f_{\text{out}}(t)$$

(5) $$\tau_{0}\frac{dq}{dt}=f_{\text{in}}\frac{E\left(f_{\text{in}}\right)}{E_{0}}-f_{\text{out}}\frac{q}{v}$$

where $E_{0}$ is the resting oxygen extraction fraction. Two other relationships are relevant. The first is the relation between oxygen extraction and flow. Buxton et al. assumed that oxygen delivery is flow-limited with a fixed capillary permeability–surface area product, which gives

(6) $$E(f)=1-{\left(1-E_{0}\right)}^{1/f}$$

Therefore, the extraction decreases as the flow increases. The second limitation is the relationship between venous outflow and venous volume. Buxton et al. adopted the empirical steady-state power law measured by Grubb et al. (22),

(7) $$f_{\text{out}}(v)=v^{1/\alpha }$$

with α≈0.38, so that 1/α≈2.63. Equations (4)–(7) provide a complete description of how neural activity drives $f_{\text{in}}$. Several variants of that relationship have been used; below, we adopt the widely used second-order form of Friston et al. (4), which is given in eq. 25 below (24, 25).

### (b) The signaling equation relating $𝚫R_{2}^{*}$ **to deoxyhemoglobin**

The BOLD signal changes mainly because of changes in the transverse relaxation rate of tissue magnetization. For a gradient-echo acquisition at echo time TE, the signal from a voxel is

(8) $$S(\text{TE})=S_{0}e^{-R_{2}^{*}\text{TE}}$$

such that a change $\Delta R_{2}^{*}$ produced by activation results in a fractional signal change

(9) $$\frac{\Delta S}{S}=e^{-\Delta R_{2}^{*}\text{TE}}-1≃-\Delta R_{2}^{*}\text{TE}\;\left(\left|\Delta R_{2}^{*}\text{TE}\right|\ll 1\right)$$

The linear approximation in (9) is well satisfied in practice. At 3T with TE = 30 ms, a 1% signal change corresponds to $\Delta R_{2}^{*}\approx -0.33\;{\text{s}}^{-1}-\Delta R_{2}^{*}\text{TE}\approx 0.01.\Delta R_{2}^{*}$ depends on separate extravascular and intravascular contributions. In the static dephasing regime around a random distribution of vessels larger than the diffusion length, the extravascular relaxation rate is proportional to the volume fraction of deoxygenated vessels multiplied by the resonant frequency offset they produce (26–28), while the total deoxyhemoglobin content is proportional to the product of venous volume and extraction:

(10) $$\Delta R_{2,E}^{*}=4.3\;\Delta \omega_{0}E_{0}V_{0}(q-1)$$

Here, $\Delta \omega_{0}=\gamma_{H}\frac{4}{3}\pi \Delta \chi_{0}\text{Hct}\;B_{0}$ is the frequency offset at the surface of a fully deoxygenated vessel with hematocrit Hct, $\gamma_{H}$ is the proton gyromagnetic ratio, and the numerical coefficient follows from Monte Carlo and analytic treatments of randomly oriented cylinders (27, 28).

The intravascular contribution arises because the transverse relaxation rate of blood itself depends on its oxygenation and varies approximately linearly with extraction over the physiological range. The venous deoxyhemoglobin concentration is q/v

(11) $$\Delta R_{2,I}^{*}=r_{0}E_{0}V_{0}\left(\frac{q}{v}-1\right)$$

where $r_{0}$ is the slope of the intravascular relaxation rate against extraction. A third term arises because blood and tissue do not have the same signal per unit volume: when the venous volume increases, blood displaces tissue, and if $\varepsilon_{r}$ is the ratio of intravascular to extravascular signal at rest, the net signal changes in proportion to $\left(1-\varepsilon_{r}\right)(1-v)$.

Collecting these contributions and applying (9) gives the signal equation in the form derived by Obata et al. (21):

(12) $$\frac{\Delta S}{S}=V_{0}\left[k_{1}(1-q)+k_{2}\left(1-\frac{q}{v}\right)+k_{3}(1-v)\right]$$

(13) $$k_{1}=4.3\;\Delta \omega_{0}E_{0}\;\text{TE},\;k_{2}=\varepsilon_{r}r_{0}E_{0}\;\text{TE},\;k_{3}=1-\varepsilon_{r}$$

Obata’s revision of the original coefficients separately revealed three physical contributions: $k_{1}$ multiplied the *total* deoxyhemoglobin content and was extravascular; $k_{2}$ multiplied the deoxyhemoglobin *concentration* and was intravascular; and $k_{3}$ multiplied the *volume* and represented the change in $S_{0}$ caused by blood displacing tissue. At 3 T with TE = 30 ms, $E_{0}=0.34,\;\varepsilon_{r}=1.43,\;r_{0}\approx 25\;{\text{s}}^{-1}$ and $\Delta \omega_{0}\approx 80.6\;{\text{s}}^{-1}$, which give $k_{1}=3.535,\;k_{2}=0.365$ and $k_{3}=-0.430$. The constants depend on the field strength and echo time (29). Later sections show that quantities used in calibrated BOLD signals follow from these three constants alone.

### (c) Blood flow, blood volume and transit time

The key to interpreting BOLD signal changes is the relationship between blood volume and blood flow. Writing CBF for cerebral blood flow and CBV for cerebral blood volume, with subscript 0 denoting the resting value throughout, the relation measured by Grubb et al. (22) during hypercapnia is

(14) $$\frac{\text{CBV}}{{\text{CBV}}_{0}}={\left(\frac{\text{CBF}}{{\text{CBF}}_{0}}\right)}^{\alpha },\;\alpha \approx 0.38$$

where α is Grubb’s exponent, a dimensionless constant.

Separately, and seemingly unrelated, the Stewart–Hamilton central volume principle states that for any compartment at steady state, the volume V, the flow F through it, and the mean transit time τ of a tracer are related:

(15) V=Fτ

Here, τ, the mean transit time (MTT), is the average time a small element of blood spends in the compartment in seconds. Equation (15) says that volume and flow are related by a single quantity with units of time, while (14) says that they are related by a dimensionless exponent. If τ is a fixed property of the vasculature, (15) will force α=1, and Grubb’s measured α=0.38 will contradict this.

τ is not a constant for resolving this apparent paradox. It is a variable property of the venous compartment and is determined by the instantaneous pressure–volume relationship. Dividing (15) for an activated state by the conditions at rest,

(16) $$\frac{\tau }{\tau_{0}}=\frac{V/V_{0}}{F/F_{0}}=\frac{v}{f}$$

and substituting Grubb’s relation (14),

(17) $$\tau (f)=\tau_{0}\;f^{\alpha -1}\;\text{or}\;\tau (v)=\tau_{0}\;v^{1-1/\alpha }$$

Grubb’s law and the central volume principle are simultaneously true but describe different properties. Equation (17) shows that Grubb’s exponent reflects the rate at which transit time decreases under vasodilation and increased flow. With $\alpha =0.38,\tau \propto f^{-0.62}$: a 60% flow increase shortens the MTT by 25%.

Considering two limits is instructive. First, α=1 would mean that transit time does not change with changing flow, corresponding to a rigid tube of fixed length whose volume tracks flow exactly, which is unphysical for a venous system of limited compliance. Second, α=0 would mean that the volume is fixed and that all of the flow change is accommodated by an increase in velocity. The measured values are between these values.

Combining equations (4) and (16), the outflow can be written as $f_{\text{out}}=v\;\tau_{0}/\tau (v)$. The model requires specifying the transit-time function τ(v), and Grubb’s law is one choice of τ(v).

### (d) Mechanical properties of the vasculature

For laminar flow through a cylindrical venule of radius r and fixed length L, Poiseuille’s law gives $F\propto \Delta Pr^{4}$, where ΔP is the pressure drop that drives flow along the vessel, while the volume satisfies $V\propto r^{2}L$. Hence, the flow relative to rest is

(18) $$f_{\text{out}}\propto \frac{\Delta P}{\Delta P_{0}}v^{2}$$

If the draining vascular bed is distensible and the downstream pressure is fixed, the driving pressure increases as the compartment inflates, which we write as $\Delta P/\Delta P_{0}=v^{\beta }$, where β≥0 is a dimensionless stiffness index (β=0 describes a pressure-clamped vessel and a larger β a stiffer vessel). Then,

(19) $$f_{\text{out}}(v)=v^{2+\beta }\;\text{and}\;\tau (v)=\tau_{0}\;v^{-(1+\beta )}$$

Comparing (19) with (17) identifies Grubb’s constant as a derived, rather than fundamental, quantity:

(20) $$\alpha =\frac{1}{2+\beta }$$

For example, β=0 gives α=0.50; Grubb’s α=0.38 corresponds to β=0.63; and a venous-specific α≈0.23 (30) corresponds to β≈2.3. The measured range of α corresponds to a physically reasonable range of venous stiffness.

### (e) An equivalent linear windkessel

An alternative viewpoint considers the vasculature to have linear compliance C (volume change per unit pressure) and a fixed downstream resistance R that does not change with activation. If p is the intracompartment venous pressure, $p_{0}$ is the resting value and $p_{v}$ is the outflow pressure, then $F_{\text{out}}=\left(p-p_{v}\right)/R$, so $f_{\text{out}}=\left(p-p_{v}\right)/\left(p_{0}-p_{v}\right)$. The compliance relation is $v-1=C\left(p-p_{0}\right)/V_{0}$. Eliminating p,

(21) $$f_{\text{out}}\left(v\right)=1+g\left(v-1\right),\;g=\frac{V_{0}}{C\left(p_{0}-p_{v}\right)}$$

where g is the normalized venous stiffness. Linearizing (19) about v=1 gives g=2+β=1/α, so Grubb’s constant is considered to be the reciprocal of the normalized stiffness of the draining bed.

### (f) Delayed compliance

The HRF is observed to possess a significant poststimulus undershoot that outlasts the flow response (31, 32), a feature that is not possible for a purely elastic balloon. Following Mandeville et al. (33), a viscous term may be added so that the outflow lag volume, with a viscoelastic time constant $\tau_{v}$ in seconds, is

(22) $$f_{\text{out}}=v^{2+\beta }+\tau_{v}\frac{dv}{dt}$$

which, combined with the balance equation (4), gives

(23) $$\frac{dv}{dt}=\frac{f_{\text{in}}-v^{2+\beta }}{\tau_{0}+\tau_{v}},\;f_{\text{out}}=f_{\text{in}}-\tau_{0}\frac{dv}{dt}$$

$\tau_{v}$ varies between inflation ($\tau_{\text{in}}$, used when $f_{\text{in}}>v^{2+\beta }$) and deflation $\left(\tau_{\text{de}}\right)$. Expanding to the first order about the resting point, writing v=1+δv and $f_{\text{in}}=1+\delta f$

$$v^{2+\beta }=(1+\delta v{)}^{2+\beta }\approx 1+(2+\beta )\delta v$$

Substituting into (23), $\frac{d\;\delta v}{dt}=\frac{\delta f-(2+\beta )\delta v}{\tau_{0}+\tau_{v}}=-\frac{\delta v}{\tau_{c}}+\frac{\delta f}{\tau_{0}+\tau_{v}}$

which is a first-order rate equation with a volume change time constant

(24) $$\tau_{c}=\frac{\tau_{0}+\tau_{v}}{2+\beta }=\alpha \left(\tau_{0}+\tau_{v}\right)$$

using α=1/(2+β) from (20).

This is another indication of what α controls the dynamic response of the vasculature.

#### Relation to previous work

The idea that the flow–volume relation should be derived from vessel mechanics rather than imposed as an empirical power law is not new, and the above discussion is a simplification and consolidation of previous literature. Mandeville et al. (33) introduced a postarteriole windkessel with delayed compliance to account for the temporal mismatch they measured between CBV and CBF in the rat cortex, and equation (22) was derived from these data. Kong et al. (34) and Zheng and Mayhew (35) developed viscoelastic windkessel models in which the steady-state Grubb power law and a nonlinear pressure–volume curve are imposed together, with the pressure–volume parameter allowed to vary dynamically so that the vessels exhibit hysteresis between dilation and contraction; Zheng and Mayhew parameterized their model by the inverse Grubb exponent, which is the quantity denoted g in (21). Boas et al. (36) computed flow and volume directly from Poiseuille’s law over an anatomically realistic vascular network. Resistance scales as $r^{4}$, while volume scales as $r^{2}$, which underlies (18), is standard in considerations of cerebrovascular physiology.

Here, we combine these suggestions into a simple formulation that shows how the single distensibility exponent β determines Grubb’s exponent through (20); that Grubb’s law is equivalent to a transit-time relation (17); and that we use these substitutions in support of the theoretical results below. We are not aware of these identities having been written down explicitly before, but the underlying ideas were previously proposed (33–36). Closed-form transfer-function solutions of linearized hemodynamic models have also been previously derived (4, 8–12), but this contribution is the first to obtain them for a lumped model whose parameters are directly physiological.

### (g) Revised balloon model equations

Collecting the results above and introducing u(t) as a transient neural excitation (a unit impulse at t=0) stimulating the BOLD response, the revised model becomes

(25) $$\frac{dz}{dt}=\varepsilon \;u(t)-\kappa \;z-\gamma \left(f_{\text{in}}-1\right),\;\frac{df_{\text{in}}}{dt}=z$$

(26) $$\frac{dv}{dt}=\frac{f_{\text{in}}-v^{2+\beta }}{\tau_{0}+\tau_{v}}$$

(27) $$f_{\text{out}}=f_{\text{in}}-\tau_{0}\frac{dv}{dt}$$

(28) $$\tau_{0}\frac{dq}{dt}=f_{\text{in}}\frac{E\left(f_{\text{in}}\right)}{E_{0}}-f_{\text{out}}\frac{q}{v}$$

together with the signal equation (12) and constants (13). In (25), which is the second-order flow-driving stage of Friston et al. (4), z is a vasoactive signal generated by neural activity with efficacy ε, decaying at rate κ (equivalently with time constant $\tau_{z}=1/\kappa$) and subject to autoregulatory feedback of strength γ (in s^−2^; for comparison with earlier work, we also report $\tau_{f}\equiv 1/\gamma$, which has units of s^2^); it is an intermediate quantity that converts a brief neural event into a smooth flow response, but only $f_{\text{in}}$ is observable. The symbol z is used here in place of s in the original formulation so that s may be reserved for the Laplace variable below. The residual system parameters are $\tau_{0}$, the resting mean transit time in seconds; β, a dimensionless stiffness index that relates volume to pressure; and $\tau_{\text{in}}$ and $\tau_{\text{de}}$, the viscoelastic time constants.

Equations (25)–(28) can be integrated numerically to predict the HRF for any set of input parameters, and this process is demonstrated in the Results section. First, we extend the model to explicitly introduce the contributions of changes in oxygen metabolism and solve the linearized system in closed form.

### (h) Oxygen metabolism as an independent drive

Optical and MRI measurements have suggested that a positive BOLD response may be preceded by brief negative deflection, the “initial dip”, attributed to oxygen consumption preceding the flow response (37, 38); moreover, the existence and magnitude of this response remain contested in gray matter (39, 40), but this response appears prominently in white matter responses (16, 41). The balloon model in its original form can produce an early negative deflection (1), but because extraction is tied to flow, it cannot produce a dip caused by an increase in metabolism that precedes the flow response. The deoxyhemoglobin change in (28) is given by $f\;E(f)/E_{0}$. The cerebral metabolic rate of oxygen (CMRO_2_) and the oxygen extraction fraction (OEF) are related as

(29) $${\text{CMRO}}_{2}=\text{CBF}\cdot \text{OEF}\cdot C_{a}$$

so that, in normalized form, $m={\text{CMRO}}_{2}/{\text{CMRO}}_{2,0}$ and $e=\text{OEF}/{\text{OEF}}_{0},m=fe$. The extraction term in (28) is therefore, by definition, normalized oxygen consumption:

(30) $$m(t)=f\frac{E(f)}{E_{0}}$$

Oxygen consumption is thereby dependent on flow. Extraction is a function of flow alone, so changes in metabolism cannot precede changes in flow, and the coupling between them is not a free parameter but is fixed by the resting extraction fraction. Any initial dip the original model produces is therefore a vascular phenomenon only, arising because the balloon must inflate before its outflow can rise so that washout of deoxyhemoglobin lags an increase in its amount. This mismatch is not a metabolic mismatch that explains this dip. The conditions under which this vascular dip occurs are given below (eqs. 55 and 56).

Allowing oxygen consumption to vary independently of flow has been considered in other analyses. Obata et al. (21) generalized the balloon model by allowing extraction to vary independently in time so that different relative time courses of CBF, CBV and CMRO_2_ produce different BOLD transients even when the flow responses are similar. Buxton et al. (23) treated CMRO_2_ as an independent input; Simon and Buxton (42) examined the dynamic dissociation between flow and BOLD; and Tang et al. (43) showed with balloon-model analyses of V1 that transient features, including differences between activations and deactivations, arise within this broader family of models. Here, we adopt the same decoupling method, producing the new results below, which include closed-form expressions for the dip (eqs. 40 and 41); an initial slope that is independent of coupling, stiffness and viscoelasticity (eq. 54); and criteria for when a dip occurs (eqs. 56 and 57).

Allowing m(t) to be independent of flow allows for more varied and realistic HRFs. There is a balance between the production of deoxyhemoglobin and the consumption of oxygen, and (28) becomes:

(31) $$\tau_{0}\frac{dq}{dt}=m(t)-f_{\text{out}}\frac{q}{v}$$

Oxygen extraction is then determined by conditions such that

(32) $$e(t)\equiv \frac{\text{OEF}}{{\text{OEF}}_{0}}=\frac{m(t)}{f_{\text{in}}(t)}$$

Equation (31) differs from (28) only in transient conditions, but there are two consequences to emphasize before further considering the overall HRF.

### (i) Calibrated fMRI and the Davis relation

The venous deoxyhemoglobin concentration is proportional to OEF, so the total deoxyhemoglobin content in the steady state is q=ve, and δq=δv+δe. Linearizing the signal equation (12) about the baseline gives $\delta S/S=-\left(k_{1}+k_{2}\right)\delta q+\left(k_{2}-k_{3}\right)\delta v$; substituting δq=δv+δe and δv=αδf,

(33) $$\frac{\delta S}{S}=-M\left[\alpha \;\delta f+\beta_{D}\;\delta e\right],\;M=V_{0}\left(k_{1}+k_{3}\right),\;\beta_{D}=\frac{k_{1}+k_{2}}{k_{1}+k_{3}}$$

M is the calibration constant of Davis et al. (18) — the maximum attainable BOLD change — and $\beta_{D}$ their field-dependent exponent. The BOLD signal is driven by a decrease in oxygen extraction, with a smaller opposing term αδf from volume dilation. Using δe=δm−δf,

(34) $$\frac{\delta S}{S}=M\left[\left(\beta_{D}-\alpha \right)\delta f-\beta_{D}\;\delta m\right]$$

Calibrated BOLD studies report measurements of the steady-state CBF–CMRO_2_ coupling ratio. This parameter describes the relation between a fractional change in flow and a fractional change in oxygen consumption n≡δf/δm; calibrated BOLD studies report values of 2–3 (19, 20). Using this simplifies to (34):

(35) $$\frac{\delta S}{S}=M\;G\;\delta m,\;G=n\left(\beta_{D}-\alpha \right)-\beta_{D}$$

G represents an overall “metabolic gain”, which vanishes at $n_{\text{crit}}=\beta_{D}/\left(\beta_{D}-\alpha \right)$; below that coupling ratio, an increase in CMRO_2_ produces a *negative* BOLD change. The sign of the metabolic contribution to resting-state BOLD signals therefore depends on whether n (the fractional change in flow per fractional change in oxygen consumption) exceeds a threshold that is itself set by α and hence by venous stiffness.

### (j) Dual pathways for stimulating a response

Neural activity can now be considered to drive two pathways that cause changes in BOLD signals: a prompt metabolic response that alters oxygen consumption and a flow response that may lag behind it by a delay Δ.

The metabolic pathway depends on m(t), the time course of the normalized rate of oxygen consumption. Neural activity does not increase oxygen consumption instantaneously. The firing of neurons uses energy supplied by increased fluxes of metabolic intermediates that involve several steps, each with its own kinetics, and for our purposes, only the aggregate effect occurs (38). We therefore describe the metabolic response to an instantaneous impulse via a simple filter constructed to match the minimal properties that are needed — the oxygen consumption starts at rest, rises in a finite time to a peak, and returns to baseline. The simplest first-order stretching of an impulse is a linear filter in which, if the quantity of interest is instantaneously displaced from its equilibrium value and returns toward it at a rate proportional to the displacement, an impulse input produces a single exponentially decaying response. The impulse response of one such stage is therefore

$$k(t)=\frac{1}{\tau_{m}}e^{-t/\tau_{m}},\;t\geq 0$$

$\tau_{m}$ = the time constant of recovery, and the response is normalized so that $\int_{0}^{\infty }k(t)dt=1$. A single stage delays and smooths its input but does not change its area. However, a single relaxation alone increases discontinuously at t=0 and decays from there, so oxygen consumption would have to change infinitely quickly at stimulus onset, which is unphysical. However, two such filters are arranged in series so that the output of the first drives the second, producing a response that matches our requirements. Writing $\tau_{m}$ for the time constant of each filter and $a_{m}$ for the gain and taking u(t) to be a unit impulse — an idealized instantaneous burst of neural activity — the result is given by the convolution of the two filter impulse responses,

$$k_{2}(t)=\int_{0}^{t}\frac{1}{\tau_{m}}e^{-\left(t-t^{\prime }\right)/\tau_{m}}\cdot \frac{1}{\tau_{m}}e^{-t^{\prime }/\tau_{m}}dt^{\prime }$$

so that

(36) $$\delta m(t)=\frac{a_{m}}{\tau_{m}^{2}}t\;e^{-t/\tau_{m}}$$

The response (36) is zero at t=0, rises to a peak at $t=\tau_{m}$ and has an area $\int_{0}^{\infty }\delta \;m\;dt=a_{m}$; thus, $a_{m}$ is the time-integrated fractional increase in oxygen consumption in units of seconds. A value of T s means that the extra oxygen consumed over the whole response equals T seconds’ worth consuming at the resting rate. More generally, for k successive stages, each time constant τ, is given $m(t)=t^{k-1}e^{-t/\tau }/\left[\tau^{k}(k-1)!\right]$, the gamma density of shape k and the scale τ—the same family of functions conventionally used to describe the measured HRF itself (5). Equation (36) is the case in which k=2; the form used for numerical integration is given in Materials and Methods.

The flow-inducing signal of (25) can be generated in the same way: in both pathways, a brief neural event is converted into a smooth, delayed physiological response by a second-order linear filter, and the two differ only in their time constants and onset delay Δ. The flow pathway is shown in (25) with the drive delay:

(37) $$\frac{dz}{dt}=\varepsilon \;u(t-\Delta )-\kappa \;z-\gamma \left(f_{\text{in}}-1\right),\;\frac{df_{\text{in}}}{dt}=z$$

### (k) Analytic description of the negative dip

Before considering the overall HRF, a useful measure of the negative dip follows from the equations above. Between 0≤t≤Δ, the flow is not increased, so $f_{\text{in}}=1$; the venous volume is constant, v=1, and dv/dt=0. In this interval tThe balloon was not allowed to inflate. From the outflow relation (27),

$$f_{\text{out}}=f_{\text{in}}-\tau_{0}\frac{dv}{dt}=1$$

Therefore, the outflow also remains the same as that at rest. Only oxygen consumption changes. The deoxyhemoglobin balance (31) becomes

$$\tau_{0}\frac{dq}{dt}=m(t)-q$$

Writing q=1+δq and m=1+δm,

(38) $$\tau_{0}\frac{d\;\delta q}{dt}+\delta q=\delta m(t)$$

This linear first-order equation is soluble. Writing $g_{0}\equiv 1/\tau_{0}$ for the washout rate constant,

$$\frac{d\;\delta q}{dt}+g_{0}\;\delta q=\frac{\delta m(t)}{\tau_{0}}$$

This gives

(39) $$\delta q(t)=\frac{1}{\tau_{0}}\int_{0}^{t}e^{-\left(t-t^{\prime }\right)/\tau_{0}}\;\delta m\left(t^{\prime }\right)dt^{\prime }$$

This is a convolution of the metabolic time course with the kernel

$$K(t)=\frac{1}{\tau_{0}}e^{-t/\tau_{0}},\;\int_{0}^{\infty }K(t)dt=1$$

K has a direct physical interpretation: it is the fraction of the deoxyhemoglobin produced at one instant that is still present in the venous compartment a time t later.

In the same delay interval v=1, the signal equation (12) simplifies to

$$\frac{\Delta S}{S}=V_{0}\left[k_{1}(1-q)+k_{2}\left(1-\frac{q}{v}\right)+k_{3}(1-v)\right]=V_{0}\left(k_{1}+k_{2}\right)(1-q)=-V_{0}\left(k_{1}+k_{2}\right)\delta q(t)$$

The volume-weighted term $k_{3}(1-v)$ vanishes because the balloon has not inflated. The BOLD signal in this window is therefore proportional to δq and negative.

Substituting the metabolic drive (36), $\delta m(t)=\left(a_{m}/\tau_{m}^{2}\right)t\;e^{-t/\tau_{m}}$, and writing $\lambda \equiv 1/\tau_{m}$ for the decay rate of the metabolic response alongside $g_{0}=1/\tau_{0}$ for the washout rate,

$$\delta q(t)=\frac{a_{m}\;g_{0}}{\tau_{m}^{2}}\int_{0}^{t}e^{-g_{0}\left(t-t^{\prime }\right)}t^{\prime }\;e^{-\lambda t^{\prime }}dt^{\prime }$$

$$\delta q\left(t\right)=\frac{a_{m}\;g_{0}}{\tau_{m}^{2}}e^{-g_{0}t}\int_{0}^{t}t^{\prime }\;e^{\mu t^{\prime }}dt^{\prime },\;\mu \equiv g_{0}-\lambda$$

By integrating by parts,

$$\int_{0}^{t}t^{\prime }e^{\mu t^{\prime }}dt^{\prime }=\frac{t\;e^{\mu t}}{\mu }-\frac{e^{\mu t}-1}{\mu^{2}}$$

$$\delta q(t)=\frac{a_{m}\;g_{0}}{\tau_{m}^{2}}\left[\frac{t\;e^{-\lambda t}}{\mu }-\frac{e^{-\lambda t}-e^{-g_{0}t}}{\mu^{2}}\right]$$

Substituting $g_{0}=1/\tau_{0}$ gives equation (40):

(40) $$\delta q(t)=\frac{a_{m}}{\tau_{m}^{2}\tau_{0}}\left[\frac{e^{-g_{0}t}-e^{-\lambda t}}{{\left(g_{0}-\lambda \right)}^{2}}+\frac{te^{-\lambda t}}{g_{0}-\lambda }\right]$$

Within the preflow window 0≤t≤Δ, equation (39) gives the deoxyhemoglobin perturbation as the convolution of the metabolic drive with the washout kernel. Suppose that oxygen consumption rises instantaneously at t=0 to a new constant level and stays there so that $\delta m\left(t^{\prime }\right)=\delta m_{0}$ for all $t^{\prime }>0$. This is the idealization in which metabolism is switched on abruptly and flow has not yet responded. Because $\delta m_{0}$ is constant, it comes outside the integral,

$$\delta q(t)=\frac{\delta m_{0}}{\tau_{0}}\int_{0}^{t}e^{-\frac{t-t^{\prime }}{\tau_{0}}}\;\text{d}t^{\prime }=\delta m_{0}\left(1-e^{-t/\tau_{0}}\right)$$

Therefore, deoxyhemoglobin accumulates toward a plateau of $\delta m_{0}$ with time $\tau_{0}$.

The minimum dip is the value reached at the moment the flow arrives, that is, at t=Δ. Applying the delay-window signal relation above,

$$\text{dip}(\Delta )={\left.\frac{\Delta S}{S}\right|}_{t=\Delta }=-V_{0}\left(k_{1}+k_{2}\right)\delta q(\Delta )=-V_{0}\left(k_{1}+k_{2}\right)\delta m_{0}\left(1-e^{-\Delta /\tau_{0}}\right)$$

For $\Delta \ll \tau_{0}$

(41) $$\text{dip}(\Delta )\approx -V_{0}\left(k_{1}+k_{2}\right)\delta m_{0}\frac{\Delta }{\tau_{0}}$$

For a given metabolic step, the depth of the dip is set by the ratio of the neurovascular delay to the mean transit time so that the measured dip constrains $\Delta /\tau_{0}$.

Equation 41 assumes an abrupt step in CMRO_2_. For the smooth drive of eq. 36, $\delta m\approx a_{m}t/\tau_{m}^{2}$ at early times, and eq. 39 gives $\delta q(\Delta )\approx a_{m}\Delta^{2}/\left(2\tau_{m}^{2}\tau_{0}\right)$ for $\Delta \ll \tau_{m},\tau_{0}$, so that the dip then grows quadratically with the delay, as found numerically below (Table 5). In both cases, the depth varies inversely with $\tau_{0}$.

### (l) An analytic form for the intermediate hemodynamic response function

The revised equations describe a linear time-invariant system and can be solved in closed form. We take a step increase in the CMRO_2_ at t=0 and allow the flow to follow with a finite rise time but initially no delay. We first derive the transfer function from CMRO_2_ to BOLD without the metabolic filter (36) that transforms neural activity into a CMRO_2_ time course or delay Δ. The response to a neural event, which contains both, is given below (eq. 59).

Writing all variables as small perturbations about rest and linearizing (26), (27), (31) and (12),

(42) $$\tau_{c}\frac{d\;\delta v}{dt}+\delta v=\alpha \;\delta f,\;\tau_{c}=\alpha \left(\tau_{0}+\tau_{v}\right)$$

(43) $$\delta f_{\text{out}}=\delta f-\tau_{0}\frac{d\;\delta v}{dt}$$

(44) $$\tau_{0}\frac{d\;\delta q}{dt}+\delta q=\delta m-\delta f_{\text{out}}+\delta v$$

(45) $$\frac{\Delta S}{S}=-A\;\delta q+B\;\delta v,\;A=V_{0}\left(k_{1}+k_{2}\right),\;B=V_{0}\left(k_{2}-k_{3}\right)$$

A is the sensitivity of the signal to deoxyhemoglobin content, and B is its sensitivity to volume.

Let s denote the Laplace variable and M(s), $F\left(s\right),V(s)$, Q(s), and $F_{\text{out}}(s)$ denote the transforms of δm,δf,δv,δq and $\delta f_{\text{out}}$, respectively. For this intermediate transfer function, the flow is taken to follow metabolism through a low-pass filter of rise time $\tau_{n}$ and gain n, where n is the steady-state CBF–CMRO_2_ coupling ratio, the ratio of the fractional change in flow to the fractional change in oxygen consumption. With these definitions,

(46) $$V(s)=\frac{\alpha F(s)}{1+\tau_{c}s},\;F_{\text{out}}(s)=F(s)\frac{1+\alpha \tau_{v}s}{1+\tau_{c}s}$$

(47) $$Q(s)=\frac{M(s)-F_{\text{out}}(s)+V(s)}{1+\tau_{0}s},\;F(s)=\frac{n\;M(s)}{1+\tau_{n}s}$$

The collection terms give the hemodynamic transfer function from CMRO_2_ to BOLD:

(48) $$H(s)=\frac{-AN(s)+B\alpha n\left(1+\tau_{0}s\right)}{\left(1+\tau_{0}s\right)\left(1+\tau_{n}s\right)\left(1+\tau_{c}s\right)}$$

(49) $$N(s)=\left(1+\tau_{n}s\right)\left(1+\tau_{c}s\right)-n(1-\alpha )-n\alpha \tau_{v}s$$

For a unit step in CMRO_2_, M(s)=1/s, and partial fractions give one exponential per root. Indexing the three roots by i with time constants $\tau_{i}\in \left\{\tau_{0},\tau_{n},\tau_{c}\right\}$,

(50) $$\Delta S(t)=p_{0}+\sum_{i}R_{i}\;e^{-t/\tau_{i}}$$

(51) $$R_{i}=\frac{-\tau_{i}^{2}P\left(-1/\tau_{i}\right)}{\prod_{j\neq i}\left(\tau_{i}-\tau_{j}\right)},\;P\left(s\right)=-A\;N(s)+B\alpha n\left(1+\tau_{0}s\right)$$

with $p_{0}=P(0)$ and $\sum_{i}R_{i}=-p_{0}$ since the response must start from zero. The HRF proper — the impulse response — is the derivative,

(52) $$h(t)=-\sum_{i}\frac{R_{i}}{\tau_{i}}e^{-t/\tau_{i}}$$

a sum of decaying exponentials. Empirical models of the HRF instead assume a functional form, such as gamma or other basis functions (5, 6); here, the form is derived, and each time constant has a physical meaning.

Three specific results can be derived from the transfer function

First, letting s→0 in (48),

(53) $$\Delta S(\infty )=n[A(1-\alpha )+B\alpha ]-A=M\left[n\left(\beta_{D}-\alpha \right)-\beta_{D}\right]$$

where $M=V_{0}\left(k_{1}+k_{3}\right)$ and $\beta_{D}=\left(k_{1}+k_{2}\right)/\left(k_{1}+k_{3}\right)$. This is algebraically identical to the calibrated BOLD relation of Davis et al. (18) obtained as an output of the model rather than as an assumption. The numerical results are given in the Results section.

Second, for $\tau_{n}>0$,

(54) $${\left.\frac{d\;\Delta S}{dt}\right|}_{0^{+}}=-\frac{A}{\tau_{0}}=-\frac{V_{0}\left(k_{1}+k_{2}\right)}{\tau_{0}}<0$$

This does not depend on n,α, or $\tau_{v}$. If CBF takes any time at all to rise — even with zero delay — the BOLD response must begin downward because deoxyhemoglobin is being produced at $t=0^{+}$ while venous washout has not yet changed. Neurovascular delay can produce a dip but is not necessary.

Third, if there is a simultaneous step $\left(\tau_{n}=0\right)$, the initial slope is

(55) $${\left.\frac{d\;\Delta S}{dt}\right|}_{0^{+}}=\frac{\alpha \left[\tau_{0}(Bn-A)+A\tau_{v}(n-1)\right]}{\tau_{0}\;\tau_{c}}$$

which is also negative — that is, a dip occurs without a delay or a flow rise time — if

(56) $$n<n_{\text{dip}}\equiv \frac{A\left(\tau_{0}+\tau_{v}\right)}{\tau_{0}B+A\tau_{v}}=\frac{\tau_{0}+\tau_{v}}{\tau_{v}+(B/A)\tau_{0}}$$

The interpretation is that the balloon must inflate before its outflow can rise, so washout of deoxyhemoglobin necessarily lags production. Combining (56) with the requirement that the plateau (53) be positive, $n>\beta_{D}/\left(\beta_{D}-\alpha \right)$, gives the window within which a transient dip is followed by a positive response,

(57) $$\frac{\beta_{D}}{\beta_{D}-\alpha }<n<n_{\text{dip}},\;\text{satisfied}\;\text{only}\;\text{if}\;\frac{\tau_{v}}{\tau_{0}}<\frac{1}{\alpha }-1=1+\beta$$

a Relationships between the presence of a dip, venous viscoelasticity and neurovascular coupling. The simplification in (57) follows from an exact identity between the signal constants,

(58) $$\beta_{D}\left(1-\frac{B}{A}\right)=\frac{k_{1}+k_{2}}{k_{1}+k_{3}}\cdot \frac{\left(k_{1}+k_{2}\right)-\left(k_{2}-k_{3}\right)}{k_{1}+k_{2}}=\frac{k_{1}+k_{3}}{k_{1}+k_{3}}=1$$

such that $B/A=1-1/\beta_{D}$ exactly and the calibration exponent cancels: a transient dip followed by a positive response requires that the viscoelastic time constant be shorter than (1+β) times the mean transit time.

### (m) Complete hemodynamic response to a neural event

Above, we described the effect of a step in oxygen consumption on the signal. The expression of the metabolic filter that relates neural impulses to changes in metabolism and the effects of delayed flow are both linear, so they can be combined with the intermediate transfer function, and an expression for the complete chain, from a neural impulse to a BOLD signal, can be derived. With $M(s)=a_{m}/{\left(1+\tau_{m}s\right)}^{2}$ for the metabolic branch and $F(s)=\varepsilon \;e^{-\Delta s}/\left(s^{2}+\kappa s+\right.\gamma )$ for the delayed flow branch, substitution into (46) and (47) gives

(59) $$H(s)=\frac{-A\;a_{m}}{{\left(1+\tau_{m}s\right)}^{2}\left(1+\tau_{0}s\right)}+\frac{\varepsilon \;e^{-\Delta s}\left[B\alpha \left(1+\tau_{0}s\right)+A\left(1-\alpha +\alpha \tau_{v}s\right)\right]}{\left(s^{2}+\kappa s+\gamma \right)\left(1+\tau_{c}s\right)\left(1+\tau_{0}s\right)}$$

The first term is the metabolic branch acting alone; it has a double root at $s=-1/\tau_{m}$ and a simple pole at $s=-1/\tau_{0}$ and inverts to a combination of $t\;e^{-t/\tau_{m}},e^{-t/\tau_{m}}$ and $e^{-t/\tau_{0}}$. The second term carries the factor $e^{-\Delta s}$, which is a time shift, so it contributes nothing to t<Δ. The initial dip is therefore the first term acting alone.

The flow term depends on the roots of $s^{2}+\kappa s+\gamma$. For practical values, these are complex $\left(\kappa^{2}<4\gamma \right)$, so the flow term is a damped oscillation rather than a simple decay. For the parameters fitted below, the damping ratio $\kappa /\left(2\gamma^{1/2}\right)$ is 0.6 to 0.9, so the oscillation is damped, and any undershoot it produces in the flow itself is modest. The impulse response is thus

(60) $$h(t)=h_{m}(t)+h_{f}(t-\Delta )\theta (t-\Delta )$$

with θ the unit step. Both parts can be written out explicitly. Inverting the first term of (59),

(61) $$h_{m}(t)=-A\;a_{m}\left[\frac{\tau_{0}\left(e^{-t/\tau_{0}}-e^{-t/\tau_{m}}\right)}{{\left(\tau_{m}-\tau_{0}\right)}^{2}}+\frac{t\;e^{-t/\tau_{m}}}{\tau_{m}\left(\tau_{m}-\tau_{0}\right)}\right]$$

The second term has roots at $s=-1/\tau_{0},s=-1/\tau_{c}$ and the conjugate pair $s=-\sigma \pm i\omega_{d}$, where σ=κ/2 and $\omega_{d}=\sqrt{\gamma -\kappa^{2}/4}$. Its inverse is two exponentials and a damped cosine of amplitude $R_{d}$ and phase ϕ,

(62) $$h_{f}(t)=R_{0}\;e^{-t/\tau_{0}}+R_{c}\;e^{-t/\tau_{c}}+R_{d}\;e^{-\sigma t}\text{cos}\left(\omega_{d}t+\phi \right)$$

Writing $c_{0}=B\alpha +A(1-\alpha )$, $c_{1}=\alpha \left(B\;\tau_{0}+A\tau_{v}\right)$ and $P=\varepsilon /\left(\tau_{c}\;\tau_{0}\right)$, the residues are

(63) $$R_{0}=\frac{P\left(c_{0}-c_{1}/\tau_{0}\right)}{\left(\tau_{0}^{-2}-\kappa \tau_{0}^{-1}+\gamma \right)\left(\tau_{c}^{-1}-\tau_{0}^{-1}\right)},\;R_{c}=\frac{P\left(c_{0}-c_{1}/\tau_{c}\right)}{\left(\tau_{c}^{-2}-\kappa \tau_{c}^{-1}+\gamma \right)\left(\tau_{0}^{-1}-\tau_{c}^{-1}\right)}$$

(64) $$R_{p}=\frac{P\left(c_{0}+c_{1}p\right)}{2i\omega_{d}\left(p+\tau_{c}^{-1}\right)\left(p+\tau_{0}^{-1}\right)},\;p=-\sigma +i\omega_{d},\;R_{d}=2\left|R_{p}\right|,\;\phi =\text{arg}R_{p}$$

Equations (60)–(64) are the complete analytic hemodynamic response functions of the model, which are the sum of three exponentials and one damped cosine, with the flow contribution switched on at t=Δ. Evaluated for the parameters of Table 4, they give

(65) $$h_{m}(t)=-2.176\left(e^{-t/1.699}-e^{-t/2.985}\right)-0.552\;t\;e^{-t/2.985}$$

(66) $$h_{f}\left(t\right)=17.803\;e^{-t/1.699}-1.699\;e^{-t/3.819}+21.052\;e^{-0.4459t}\text{cos}(0.2105\;t-2.442)$$

in units where $V_{0}$ is factored out of A and B, with ε=0.50 as set by the peak-CBF constraint. The damped cosine in (66) has a period $2\pi /\omega_{d}$ of 29.8 s but decays with a time constant 1/σ of 2.2 s, so it does not appear as ringing. Its negative half-cycle falls within the first 4 s, where it offsets the large positive washout term, and it is positive during the undershoot; the undershoot is instead produced mainly by the negative balloon-relaxation term in $\tau_{c}$ and by the tail of the metabolic branch (see Fig. 7). Plots of the HRF are provided below.

## Numerical and analytical results

The theoretical derivations provide analytic expressions for the HRF and allow the HRF to be derived numerically and fitted to the canonical HRF. The results below are all numerical: the revised model equations were first integrated and fitted as described in the Materials and Methods. No new experimental measurements are reported; the target for fitting is the canonical HRF used in standard fMRI analysis software, and comparisons are made with physiological values from the literature. The analytic expressions are also illustrated for different conditions and choices of physiological parameters and are shown to be consistent with the other results.

### (i) Fitting the revised equations to the canonical HRF

Equations (25)–(28) with the signal equation (12) were fitted to the SPM canonical HRF at 3 T (Table 1, Fig. 1). Two physiological constraints were imposed as penalties on the objective function so that the fitted parameter set reproduces the canonical shape while also operating at a realistic response amplitude with physically admissible vessel mechanics.

First, the peak increase in CBF is held at +40%. Second, the venous stiffness index is required to satisfy β≥0 since a negative β represents a vessel whose pressure falls as it inflates. With a signal constant of (13) at 3 T and a resting venous blood volume fraction $V_{0}=0.03$, a +40% peak CBF increase produces a peak BOLD change of 0.63%, which lies in the 0.5–2% range typical for evoked responses at this field strength and echo time. Left unconstrained, the fit would drive the neural efficacy ε toward zero and return a parameter set that describes the curve well but corresponds to a vanishingly small physiological response.

A fitted α of 0.50 lies above Grubb’s 0.38, and a fitted $\tau_{0}$ of approximately 4.5 s exceeds the 2 s conventionally assumed.

Table 2 lists the physiological quantities predicted at this operating point, and Fig. 2 shows their time courses during the hemodynamic response. The venous volume peaks 1.8 s after flow and returns far more slowly; the mean transit time falls from 4.55 s to 4.02 s and then overshoots. The +13% CBV response to a +40% CBF response corresponds to an effective α of approximately 0.37 measured dynamically, below the static value of 0.50, because the balloon never reaches a steady state during a brief event.

The model is nonlinear in drive amplitude: the relative undershoot shrinks from −0.13 to −0.02 as the drive increases sixteenfold (Fig. S1), which is one reason why a single canonical HRF cannot be corrected across stimulus intensities (7, 23).

#### Comparison of the analytic expression with the canonical HRF

Equations (61)–(64) give h(t) explicitly. The canonical HRF contains no initial dip, so for comparison, the metabolic gain was set to $a_{m}=0$; the response then decreased to the flow branch (62) alone, a sum of two exponentials and a damped cosine.

The three-term expression reproduces the canonical response with $R^{2}=0.998$ (Table 3, Fig. 3), the largest residual being 5.8% of the peak and confined to the first two seconds. The fitted peak falls at 4.94 s against the canonical 5.00 s, and the undershoot reaches −0.082 against −0.089. This result is a closer match than the numerically integrated nonlinear model ($R^{2}=0.994$, Table 1).

The parameters returned are physiologically reasonable and were obtained independently of those in Table 1 from the linearized solution rather than from the nonlinear integration. The transit time $\tau_{0}=1.97\;\text{s}$ is close to the 2 s conventionally assumed and considerably shorter than the 4.55 s of the integrated fit; the stiffness index again goes to β=0, giving α=0.50; and the damped cosine has $\sigma =0.406\;{\text{s}}^{-1}$ and $\omega_{d}=0.316\;\text{rad}\;{\text{s}}^{-1}$, a period of 19.9 s. Because 1/σ=2.5s is much shorter than the period, the oscillation is heavily damped and does not appear as ringing. At the undershoot minimum (15 s), essentially all of the negative signals originate from the balloon relaxation term in $\tau_{c}$ (−0.088 of the peak), with the damped cosine contributing −0.002 and the washout term +0.009; thus, the undershoot here reflects delayed compliance rather than oscillatory flow.

Using the constrained parameters of Table 1 ($\tau_{0}=4.55\;\text{s},\tau_{v}=7.52\;\text{s},\alpha =0.5$; hence, $\tau_{c}=6.04\;\text{s}$, and n=2.2) and a simultaneous step in flow $\left(\tau_{n}=0\right)$, the step response (50) reduces to a two-term exponential,

(67) $$\frac{\Delta S(t)}{\delta m}=0.03792-0.14039\;e^{-t/4.549}+0.10247\;e^{-t/6.036}$$

The corresponding impulse response changes sign at t=11.0s, the onset of the poststimulus undershoot. The plateau of (67) is 3.792% per unit relative CMRO_2_ step, identical to the value returned by the Davis relation (35) with M=0.0932 and $\beta_{D}=1.256$, confirming (53).

For the metabolism-first parameters of Table 4 $\left(\tau_{0}=1.70\;\text{s},\tau_{v}=\tau_{\text{de}}=10.03\;\text{s}\right)$, the zero-delay threshold from (56) is $n_{\text{dip}}=1.13$, which is well below the steady-state coupling n=3.80 (and the peak ratio $n_{\text{peak}}=5.97$); thus, without a neurovascular delay, no dip occurs, consistent with the first row of Table 5.

The impulse responses obtained from (52) are shown in Fig. 4. With a finite flow rise time, the initial slope is $-A/\tau_{0}=-2.57\%$ per second per unit CMRO_2_ step, independent of n,α and $\tau_{v}$, as required by (54); the resulting dips are −0.52%, −0.98% and −1.74% of the step for rise times of 0.5, 1 and 2 s, respectively, with nadirs at 0.49, 0.92 and 1.66 s, respectively (Fig. 5). For the Table 1 parameters, the two thresholds in (57) are 1.661 and 1.429, so the window is closed, and a simultaneous step cannot produce a transient dip; the window opens once $\tau_{v}<(1+\beta )\tau_{0}=4.55\;\text{s}$.

Both analytic solutions were verified against direct numerical integration of the full nonlinear equations. Linearization is reliable for calculating the few percent of CMRO_2_ changes typical of resting-state fluctuations but should be used with care for strong evoked responses.

Equation (52) expresses the HRF as a sum of three decaying exponentials, one for each time constant of the system, and Fig. 6 plots those terms separately together with their total for a representative flow rise time of $\tau_{n}=1\;\text{s}$. The individual terms are much larger than the response they produce: the largest residue is 5.6 times the plateau. The venous washout term in $\tau_{0}$ is positive, and the flow and balloon terms are negative; thus, the sign of early response is set such that the patient decays fastest. With a finite flow rise time, the flow term dominates at $t=0^{+}$, and the response begins at $-A/\tau_{0}=-2.57\%$ per second, as (54) requires that the washout term then reaches a positive peak of 0.93% at 2.69 s, and the slowest term, the balloon relaxation in $\tau_{c}=6.04\;\text{s}$, lasts longer and produces the crossing back through zero at 15.4 s, which becomes the poststimulus undershoot.

The same decomposition applied to the complete response to a neural event is shown in Fig. 7. Equations (65) and (66) are the numerical forms of the two branches at the metabolism-first operating point of Table 4, and each is a sum of three terms: for the metabolic branch, two exponentials and the $t\;e^{-t/\tau_{m}}$ term; for the flow branch, two exponentials and the damped cosine. The right panel shows how the two branches combine: the metabolic branch acts alone for t<Δ and is wholly responsible for the dip, −0.063 at 1.23 s; the delayed flow branch then dominates, producing a peak of 0.776 at 4.80 s; and the undershoot of −0.042 at 15.5 s arises from the viscoelastic balloon-relaxation term of the flow branch (−0.040) and the slowly decaying tail of the metabolic branch (−0.036), partly offset by the damped cosine (+0.029), which is positive at that time. Each of the three features of the HRF is thus attributable to an identifiable term of the analytic solution.

### (ii) Calibration constants derived from the model

The constants that are normally assumed to calibrate BOLD signals are derived and may be compared with the values conventionally adopted.

The derived exponent $\beta_{D}=\left(k_{1}+k_{2}\right)/\left(k_{1}+k_{3}\right)=3.900/3.105=1.256$ may be compared with the value of approximately 1.3 conventionally assumed at 3 T in calibrated BOLD (18–20, 44).

With $\alpha =0.50,\beta_{D}=1.256$ and n=2.2, the metabolic gain is G=0.407, and the sign-reversal threshold is $n_{\text{crit}}=1.66$.

### (iii) Fitting an HRF with an initial dip

The metabolism-first formulation (eqs. 26, 27, 31, 36 and 37) was fitted to the canonical HRF with an early negative lobe added, scaled so that the nadir was −12% of the positive peak, under the same +40% peak-CBF constraint and with the added physical requirement that OEF never exceeds unity. All ten parameters were refitted (Table 4; response features in Table S3). The dip nadir occurs at 1.30 s, the positive peak occurs at 4.94 s, and the late undershoot occurs at 15.2 s (Figure 8). Oxygen extraction increases from 0.340 to 0.356 during the delay and then falls to 0.260 once the flow arrives (Fig. 9).

Allowing metabolism to increase ahead of flow reduces $\tau_{0}$ from 4.55 s down to 1.70 s and raises β from 0 to 1.07 so that the implied Grubb exponent falls from 0.50 to 0.326. Both revised values are closer to the independent measurements — a venous MTT of approximately 2 s and a venous-specific α of 0.2–0.4 (30) — than are those in Table 1. In these fits, tying oxygen consumption to flow biased the estimated transit time and flow–volume exponent upward. The two coupling ratios in Table 4 differ because the flow and metabolic responses have different shapes and onset times.

Holding all the other parameters fixed and varying only the flow delay Δ gives the relations in Table 5 (Fig. S3). The nadir latency tracks the delay almost exactly, $t_{\text{dip}}\approx \Delta +0.1\text{s}$, so the timing of the dip is a direct readout of the neurovascular delay. The dip depth grows quadratically at small Δ and closer to linear thereafter, so a measured depth of 10% implies Δ≈1.0s, and 20% implies Δ≈1.7s. The positive peak is itself delayed approximately one-for-one with Δ at 1.1 s per second, so that fitting a fixed canonical HRF across regions with different neurovascular delays will misestimate both latency and amplitude. This difference may be related to the reduced magnitudes, delayed onset and prolonged initial dips reported in white matter tracts relative to the cortex (16, 41).

## Discussion

We have derived explicit analytic expressions for the HRF of a lumped balloon model of the BOLD effect, requiring only linearization about rest. Three results should be emphasized.

First, Grubb’s exponent is not an independent property of the vasculature. Equation (17) shows that the Grubb relationship describes how the mean transit time of blood shortens when flow increases, and (20) shows that within a simple mechanical picture, it is fixed by a single distensibility exponent. Thus, the quantities that actually modulate the hemodynamic response are $\tau_{0}$, which is directly measurable, and the stiffness of the draining vessels, a property that may differ between gray and white matter, between brain regions, and with age or vascular disease.

Second, the source term in the previous balloon model was the normalized CMRO_2_, by which oxygen consumption changes in parallel with flow. The original balloon model can still produce an early negative deflection, but this deflection is a vascular transient rather than the metabolic mismatch that the initial dip is considered to signify. Replacing that term with an independently specified term m(t), as in earlier generalizations (21, 23), does not affect steady-state values but changes the transient response, and the analytic solution makes the consequences explicit: with any finite flow rise time, the response must begin downward with a slope $-V_{0}\left(k_{1}+k_{2}\right)/\tau_{0}$ that is independent of coupling, stiffness and viscoelasticity (54); even with a simultaneous step, a dip occurs whenever $n<n_{\text{dip}}$ (56), simply because the balloon must fill before its outflow can rise. Whether the dip is detectable at conventional field strengths is a separate question (39, 40).

Third, the reformulation returns the calibrated BOLD relations as outputs. Equation (53), derived by taking s→0 in the transfer function, is algebraically the Davis relation, and $\beta_{D}=1.256$ follows from the Obata signal constants alone rather than being assumed.

An immediate application of our revised model is to tissues in which the HRF is known to differ from that of the cortex. BOLD signals are reliably detected in white matter (15, 17), where HRF has a reduced magnitude, delayed onset and a more prominent initial dip and varies in shape along and between tracts (16, 41, 45). Within the present framework, these are not independent observations: a longer neurovascular delay lengthens the dip and delays the peak (Table 5), while a smaller venous blood volume fraction is expected from the lower microvessel density of white matter (46), and a longer transit time reduces the amplitude and slows the response. White matter also has a distinct energy budget (47), and its BOLD responses are modulated by myelination (48), effects that could affect m(t) and n.

The origin of the poststimulus undershoot has not been clarified (31, 32). The present model arises mainly from delayed compliance, the mechanism proposed by Mandeville et al. (33) and developed by Kong et al. (34) and Zheng and Mayhew (35). Blockley et al. (49) reported that explaining their undershoot in this way required blood volume to take more than 55 s to return to baseline after a 4.8 s stimulus, which they considered implausible, and discussed a poststimulus increase in oxygen metabolism as an alternative; poststimulus neuronal activity may also contribute to this change (50). The fits reported here do not require slow volume recovery; after a brief event, the venous volume in the numerical solutions shown in Tables 1 and 4 returns to within 10% of its peak excursion within 17 to 18 s, consistent with linearized volume time constants $\tau_{c}$ of 6.0 and 3.8 s, respectively. Moreover, in the metabolism-first solution, the tail of the metabolic branch, that is, the CMRO_2_ that remains elevated after flow has returned, contributes −0.036 to the undershoot, comparable to the −0.040 from balloon relaxation (Fig. 7). The two mechanisms are therefore not mutually exclusive.

## Limitations

A single compartment represents venous, capillary and arterial volume changes. The arterial CBV responds rapidly, whereas the venous CBV changes more slowly (51), allowing the arterial volume to vary and improving the fit with contrast agent data (49); moreover, layer-specific VASO reveals substantial capillary and arteriolar volume changes (52). Arterial contributions to the total CBV response are thus well established (30, 36). The single compartment is therefore an effective surrogate description appropriate for modeling conventional-resolution gradient-echo BOLD effects but not, in reality, a purely venous mechanical compartment. If compliant upstream volume changes are incorporated, the compartment will appear more compliant than the venous bed, which inflates the fitted $\tau_{0}$ and α and biases β downward. This may explain why the fits of Tables 1 and 3 reduce β to its lower bound. Three-compartment and multicompartment models (53, 54) could separate these contributions into more complex models.

The coupling ratio n is treated as fixed; if it varies with baseline metabolism, as some calibrated BOLD data suggest, then G is not constant, and (35) acquires an additional term. The value $\beta_{D}=1.256$ inherits the assumptions behind $\Delta \omega_{0},r_{0}$ and $\varepsilon_{r}$ at 3 T and is a consistency check rather than an independent measurement.

The metabolic drive (36) is phenomenological. Only its aggregate output enters the model, so the individual relaxations cannot be identified from BOLD data, and $\tau_{m}$ should be read as a lumped constant describing the overall delay between neural activity and oxidative flux rather than the rate of any single biochemical step.

Finally, Δ is modeled as a pure transport lag; a distributed lag would fit comparably and is arguably more physiological but has no closed-form dip expression.

## Materials and Methods

### Numerical integration.

Equations (25)–(28), or (26), (27), (31) and (37), with metabolic drive (36), for the metabolism-first formulation, were integrated with a fourth-order Runge–Kutta scheme at a time step of 20 ms over 0–32 s, with the neural drive u(t) a unit impulse at t=0. The BOLD signal was evaluated according to (12). The metabolic drive (36) was integrated in its two-stage form, $\tau_{m}\;dx/dt=-x+a_{m}u(t)$ and $\tau_{m}\;dy/dt=-y+x$, with m=1+y, which is the standard way of expressing a second-order response as a pair of first-order equations so that it can be advanced by the same solver as the balloon; x and y are internal to the integration and carry no physiological meaning.

### Fitting.

The target was the canonical HRF used in SPM, the difference between two gamma densities with shape parameters 6 and 16 and a 1:6 amplitude ratio. The model output was normalized to the unit peak and fitted by multistarting Nelder–Mead simplex minimization of the mean squared difference using 18 starting points comprising three literature-based sets and 15 drawn at random from the bounded parameter space; the best solution was subsequently refined on the fine time grid. A quadratic penalty was added to the constrained fits to enforce a peak CBF increase of +40%, and where stated, a penalty was applied to enforce OEF ≤ 1. Identifiability was assessed by profiling the cost against β and $\tau_{0}$, with all remaining parameters refitted at each point.

### Fixed constants.

MR constants were fixed at 3 T values: TE = 30 ms, $\Delta \omega_{0}=80.6\;{\text{s}}^{-1},r_{0}=25\;{\text{s}}^{-1},\varepsilon_{r}=1.43$ and $V_{0}=0.03$, giving $k_{1}=3.535,k_{2}=0.365$ and $k_{3}=-0.430$. The reference physiological values are listed in Table S2. Resting extraction was fixed at $E_{0}=0.34$ since amplitude normalization leaves it only weakly identifiable from the response shape.

### Code availability.

All analyses were performed in Python using only NumPy and Matplotlib; the Runge–Kutta integrator and Nelder–Mead optimizers were implemented directly. The code and numerical outputs are provided in *data file S1*.

## Supplementary Materials

This PDF file includes:

Figs. S1 to S3

Tables S1 to S4

The other Supplementary Materials for this manuscript include the following:

Data file S1 (code and numerical output)

## Figures and Tables

**Figure 1.**
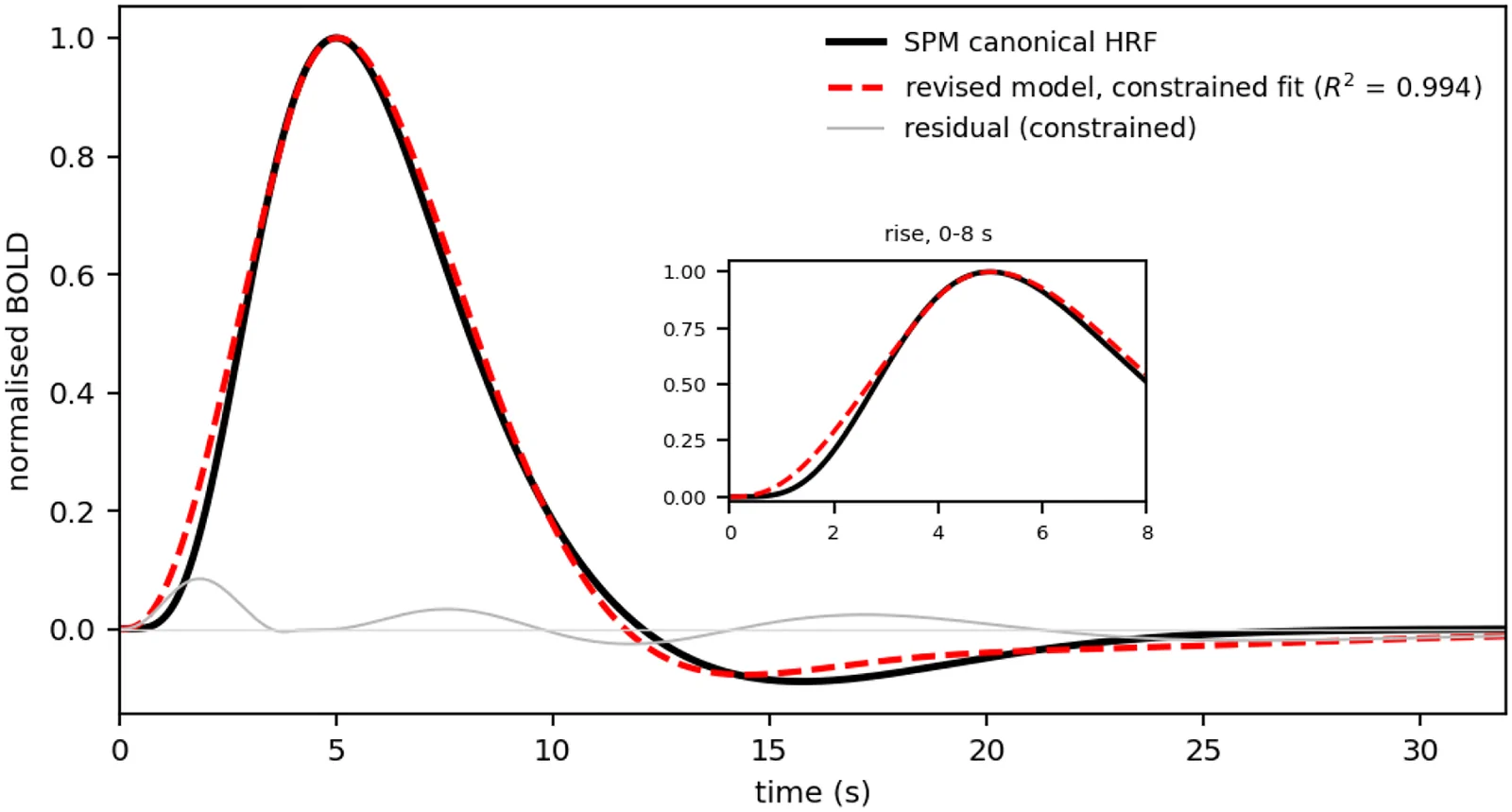
Fit of the revised model to the canonical HRF. The fitted curve (red, dashed) is the constrained solution of Table 1, with the peak CBF held at +40% and $\beta \geq 0,R^{2}=0.994$. The light gray trace is the residual, whose largest feature is a small early overshoot at 1–2 s. Inset: the rising phase, 0–8 s.

**Fig. 2.**
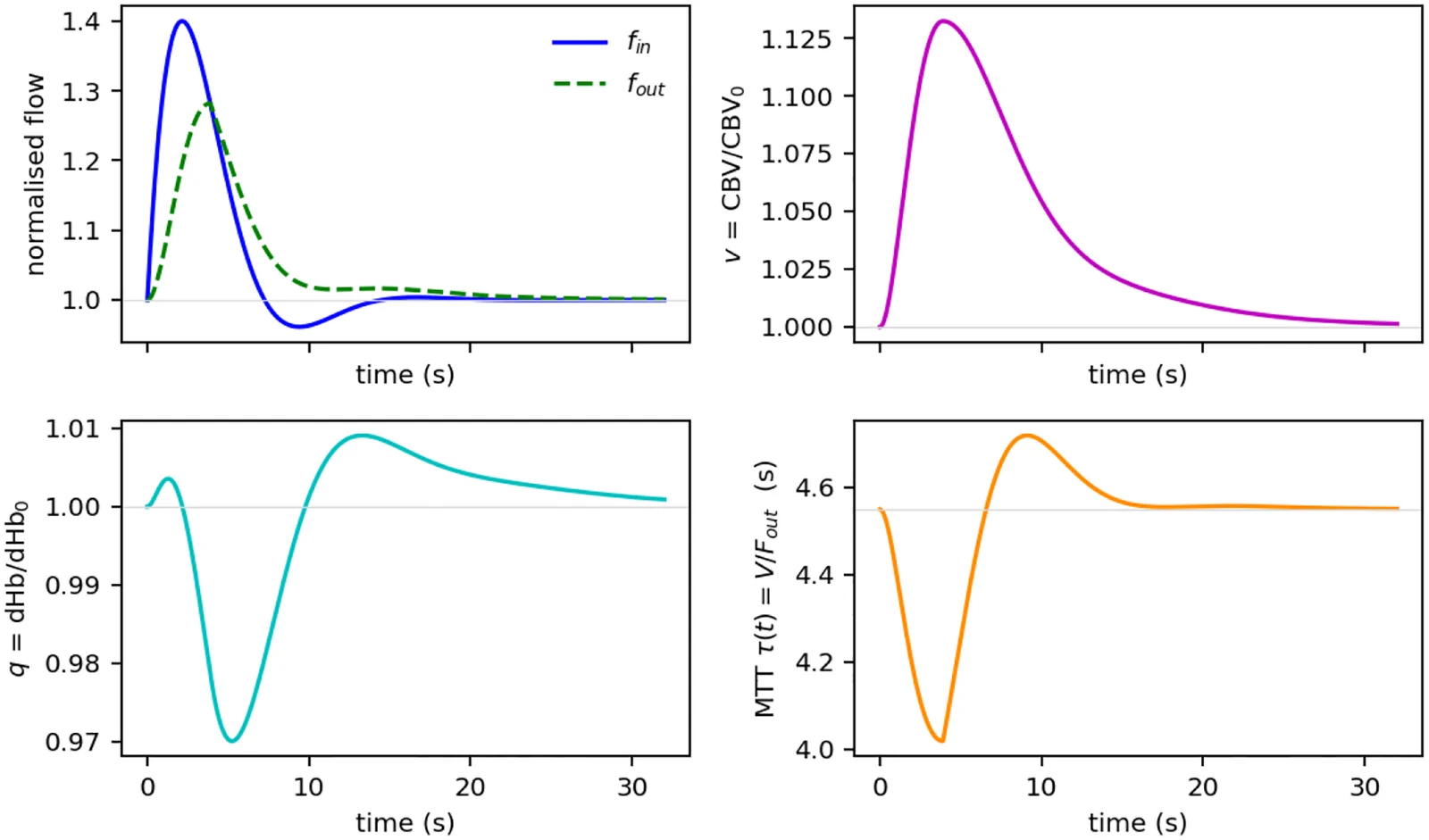
Physiological variables at the constrained operating points. (Top left) Normalized inflow and outflow; (top right) venous volume; (bottom left) total deoxyhemoglobin; (bottom right) instantaneous mean transit time $\tau (t)=V/F_{\text{out}}$.

**Fig. 3.**
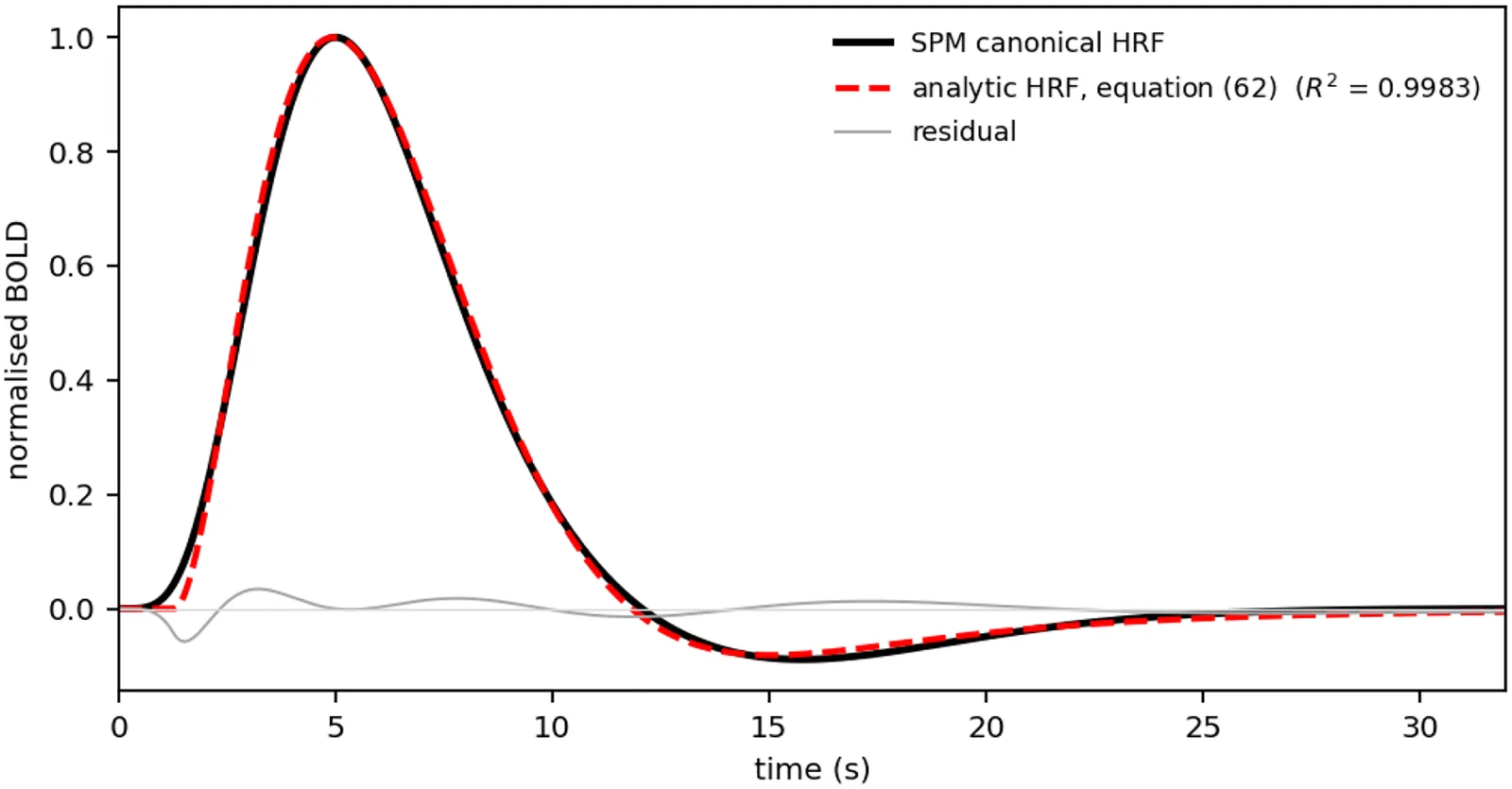
The analytic HRF was fitted to the canonical response. The closed form (62), a sum of two exponentials and a damped cosine, is fitted directly to the SPM canonical HRF (black) with the metabolic gain set to zero. The residual (gray) is largest during the first two seconds and nowhere exceeds 5.8% of the peak.

**Fig. 4.**
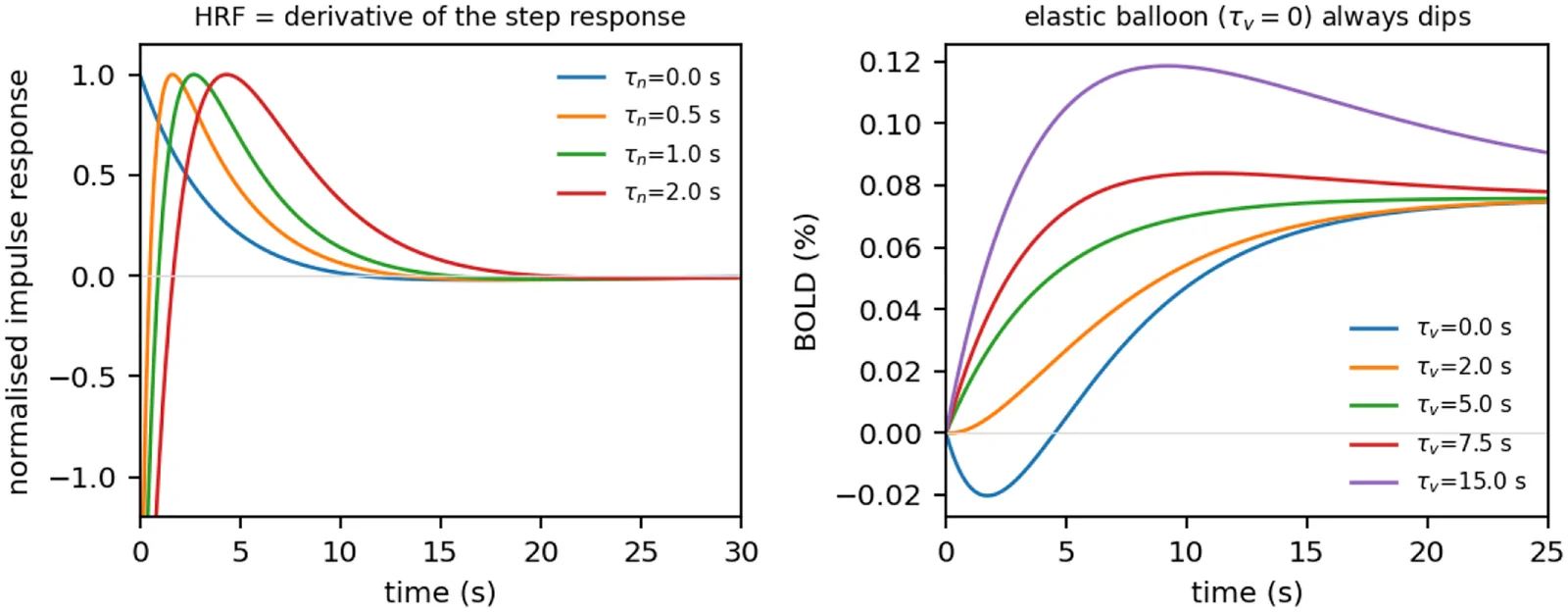
The analytic HRF for a range of flow rise times. (Left) Impulse responses from equation (52) for flow rise times $\tau_{n}$ of 0 to 2 s; with any finite rise time, the response becomes negative, as required by equation (54). (Right) Step responses showing that an elastic balloon $\left(\tau_{v}=0\right)$ produces a dip even when the CBF steps simultaneously with the CMRO2 and that the dip is progressively abolished as delayed compliance is added.

**Fig. 5.**
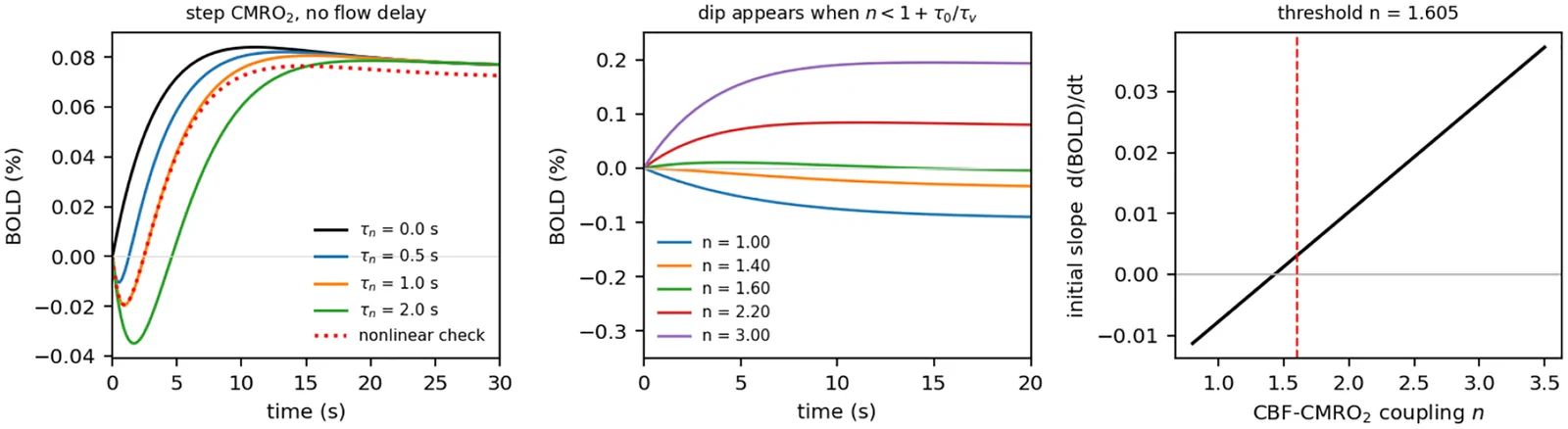
Validation of the analytic solution and the dip conditions (Left) Analytic step responses from equations (48)–(51) for flow rise times of 0–2 s, with a numerical integration of the full nonlinear model overlaid (dotted). (Center) Effect of the coupling ratio n. (Right) The initial slope changes sign at $n_{\text{dip}}=1.429$, as predicted by equation (56); the dashed line marks the value $1+\tau_{0}/\tau_{v}$ obtained if the volume term is neglected.

**Fig. 6.**
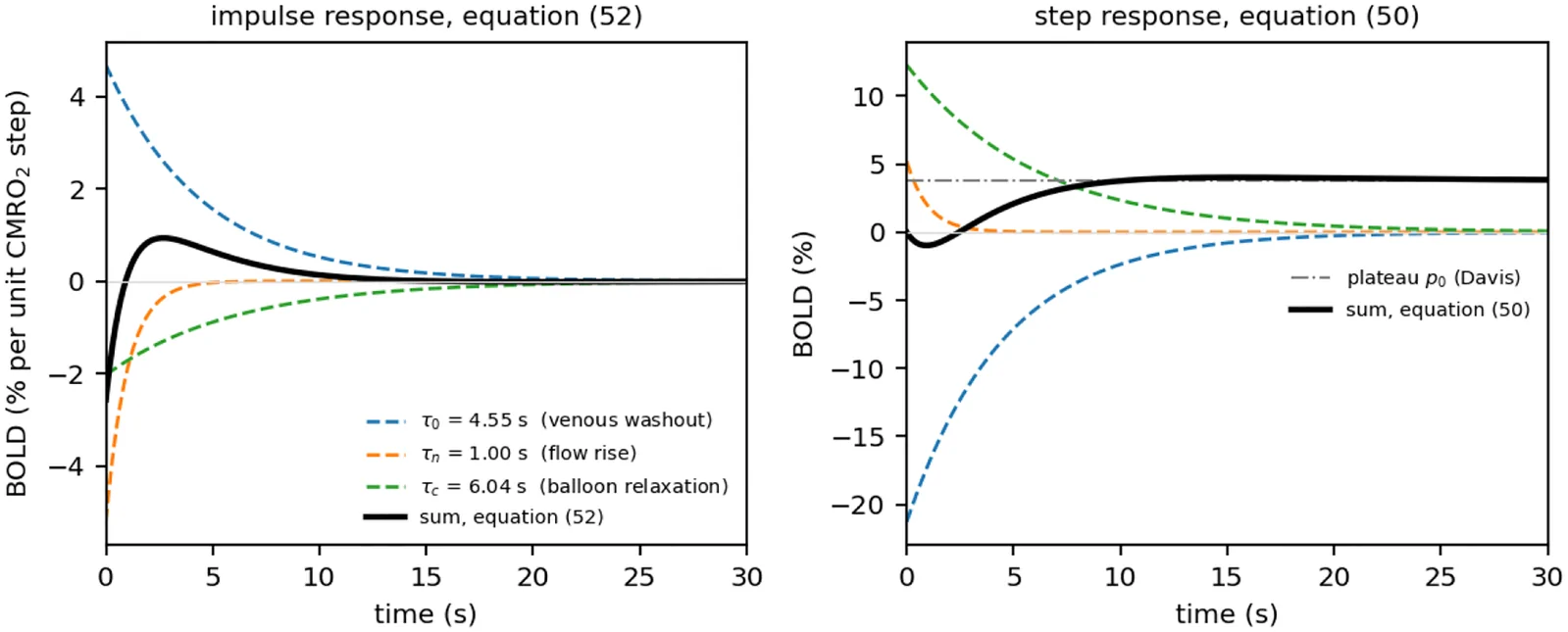
The analytic HRF is the sum of the exponentials. The terms of equation (52) (left, impulse response) and equation (50) (right, step response) are plotted separately (dashed) together with their sum (black) for the constrained parameters of Table 1 and a flow rise time $\tau_{n}=1\;\text{s}$. The colors identify the three time constants: venous washout $\tau_{0}=4.55\;\text{s}$, flow rise $\tau_{n}=1\;\text{s}$ and balloon relaxation $\tau_{c}=6.04\;\text{s}$. The individual terms are several times larger than the response itself and cancel at t=0; the dashed gray line in the right panel is the plateau $p_{0}$, which equals the Davis value.

**Fig. 7.**
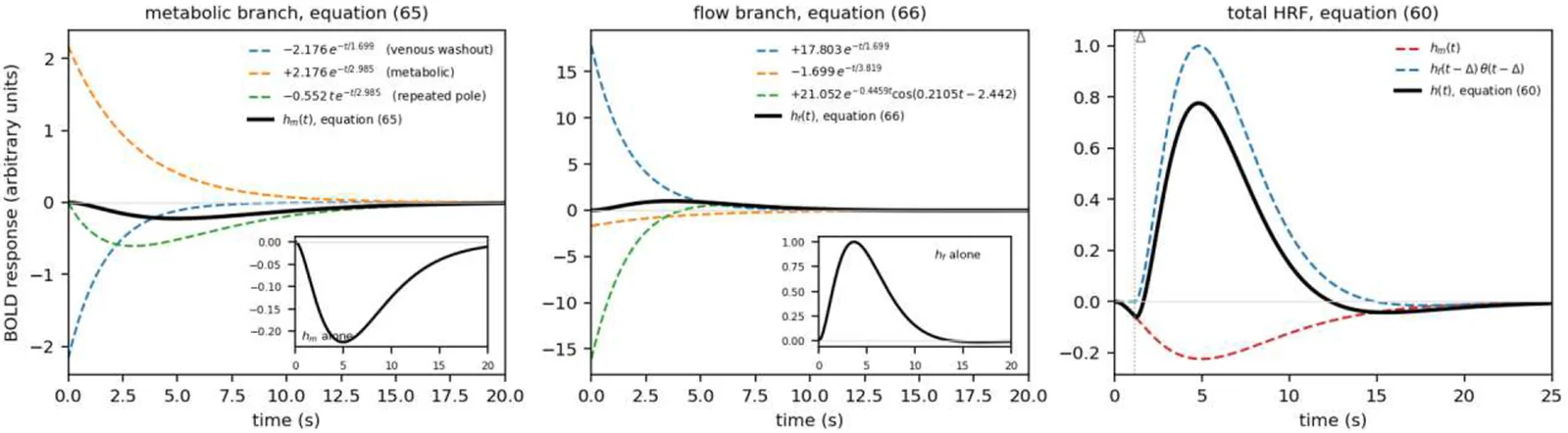
The complete HRF is the sum of the exponentials. (Left) The metabolic branch (65): two exponentials and the $t\;e^{-t/\tau_{m}}$ term from the repeated pole, with their sum $h_{m}(t)$ in black; the inset repeats the sum on its own scale. (Center) The flow branch (66): two exponentials and the damped cosine from the complex pole pair, with their sum $h_{f}(t)$; note that the individual terms reach 17.8, while the sum peaks at 1.0. (Right) The two branches combined as in (60); the flow branch delay Δ=1.15s (dotted vertical line). The metabolic branch alone produces the initial dip, the flow branch produces the peak, and the balloon-relaxation term of the flow branch and the tail of the metabolic branch produce the undershoot.

**Fig. 8.**
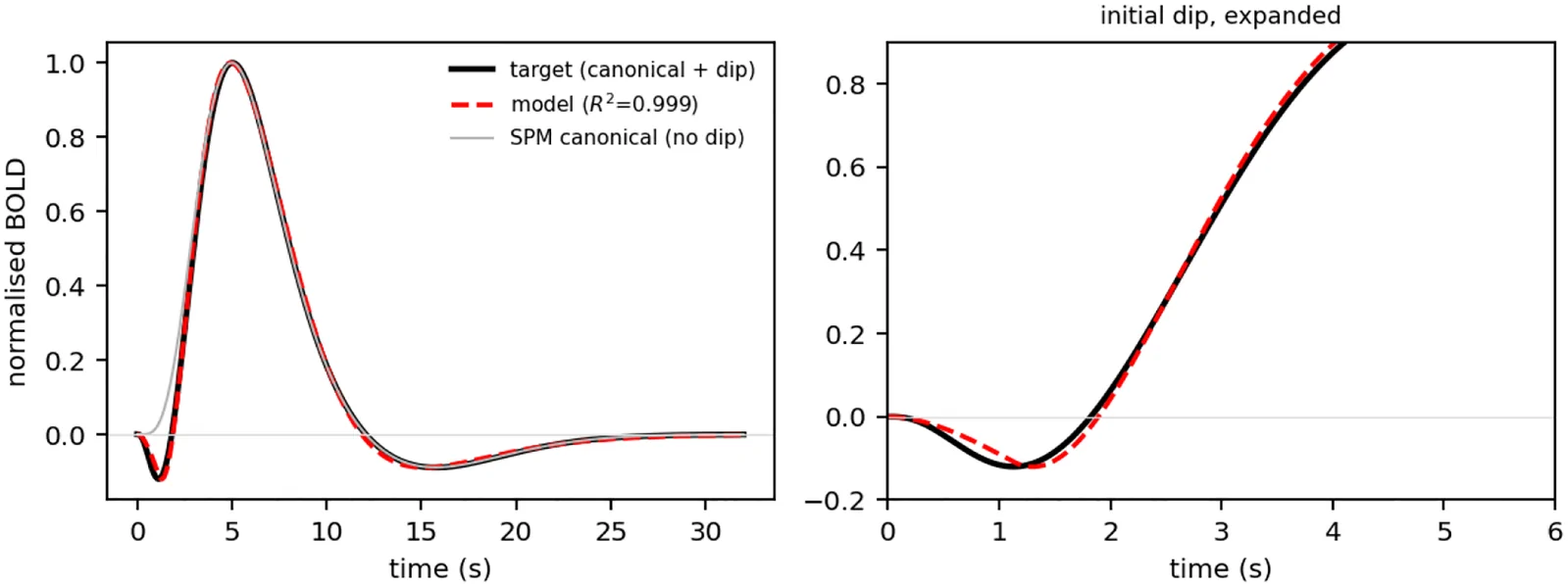
Fit of the metabolism-first model to a canonical HRF with an initial dip. The right panel shows the expansion of the first six seconds.

**Fig. 9.**
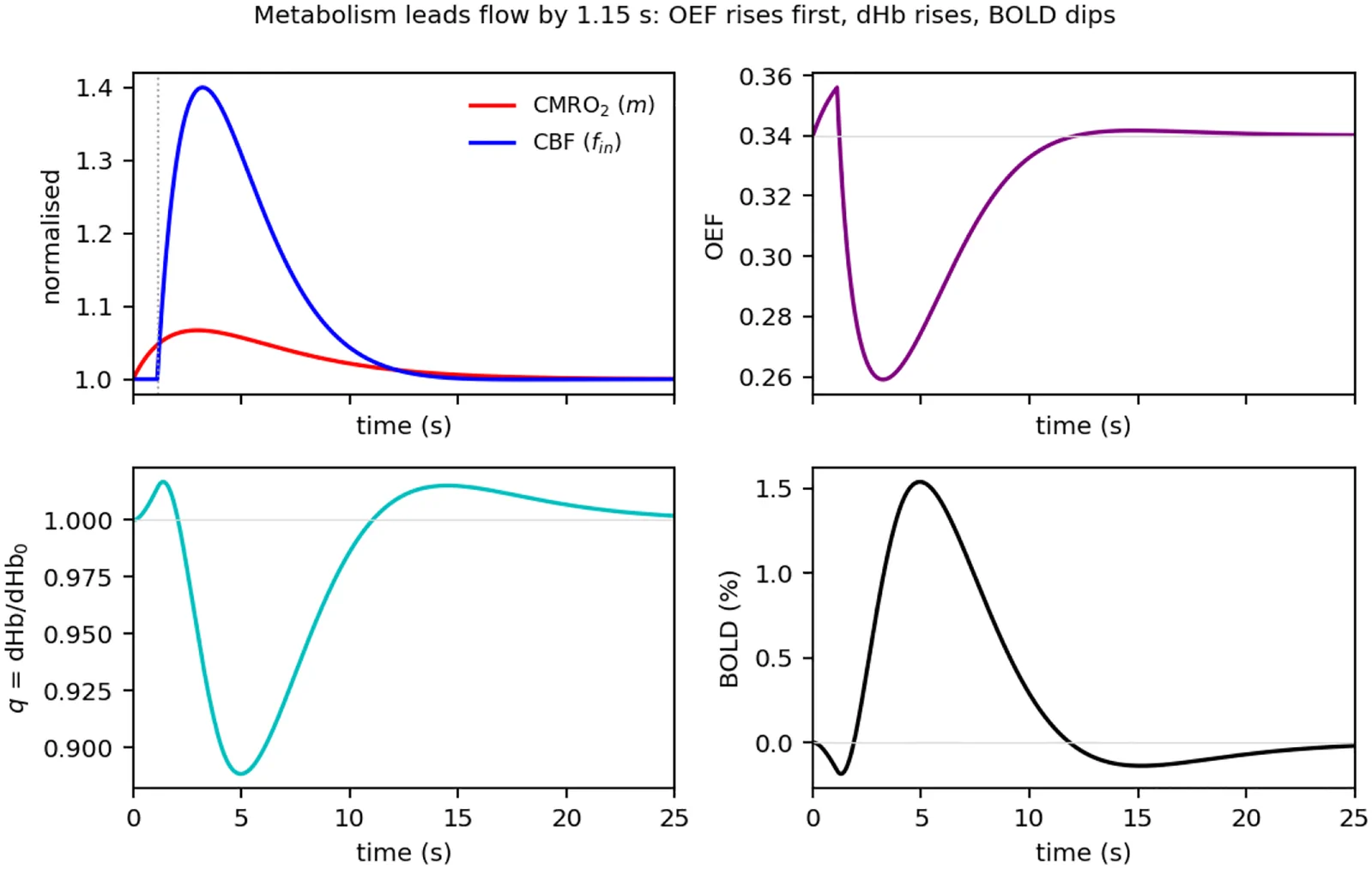
Mechanism of the initial dip. CMRO2 (red) increases 1.15 s before CBF (blue), so oxygen extraction must increase to meet demand; total deoxyhemoglobin therefore increases, and the BOLD signal becomes negative. When the flow arrives, the extraction coefficient falls to 0.26, and a familiar positive response follows.

**Table 1.** Parameters of the constrained fit to the canonical HRF.

Parameter	Symbol	Value	Interpretation
Vasoactive signal decay	$\tau_{z}=1/\kappa$	1.55	decay of z (s)
Autoregulatory feedback	$\tau_{f}=1/\gamma$	3.43	CBF feedback $\left({\text{s}}^{2}\right)$
Resting mean transit time	$\tau_{0}$	4.55	venous MTT (s)
Venous stiffness index	β	0.00 (at bound)	dimensionless
Inflation viscosity	$\tau_{\text{in}}$	0.00	s
Deflation viscosity	$\tau_{\text{de}}$	7.52	s; drives the undershoot
Neural efficacy	ε	0.43	sets response amplitude
Resting extraction	$E_{0}$	0.34 (fixed)	OEF at rest
Implied Grubb exponent	α=1/(2+β)	0.500	derived, not fitted
Fit quality	$R^{2}$	0.9945	vs canonical HRF

**Table 2.** Predicted physiology at the constrained operating point.

Quantity	Value	Comment
Peak CBF	+40%	imposed constraint
Peak CBV	+13.2%	lags CBF by 1.8 s
Minimum total deoxyhemoglobin	−3.0%	at 5.2 s
MTT at peak flow	4.02 s (−12%)	from 4.55 s at rest
Peak BOLD ($V_{0}=0.03$)	0.63%	plausible for 3 T event-related
Volume relaxation $\alpha \left(\tau_{0}+\tau_{\text{de}}\right)$	6.04 s	sets undershoot duration

**Table 3.** Parameters of the analytic HRF (62) fitted to the canonical response.

Parameter	Symbol	Value	Interpretation
Vasoactive signal decay	$\tau_{z}=1/\kappa$	1.23	decay of z (s)
Autoregulatory feedback	$\tau_{f}=1/\gamma$	3.77	CBF feedback $\left({\text{s}}^{2}\right)$
Resting mean transit time	$\tau_{0}$	1.97	venous MTT (s)
Venous stiffness index	β	0.00	dimensionless
Viscoelastic time constant	$\tau_{v}$	10.10	s; drives the undershoot
Neural efficacy	ε	0.74	sets response amplitude
Neurovascular delay	Δ	1.26	s; onset lag
Metabolic gain	$a_{m}$	0 (fixed)	no dip in the canonical HRF
Implied Grubb exponent	α=1/(2+β)	0.500	derived, not fitted
Volume relaxation	$\tau_{c}=\alpha \left(\tau_{0}+\tau_{v}\right)$	6.03	derived, not fitted
Damping of the cosine	σ=κ/2	0.406	${\text{s}}^{-1}$
Damped frequency	$\omega_{d}$	0.316	$\text{rad}\;{\text{s}}^{-1}$ (period 19.9 s)
Fit quality	$R^{2}$	0.9983	vs canonical HRF

**Table 4.** Parameters for the metabolism-first fit to a canonical HRF with a 12% initial dip.

Parameter	Symbol	Value	Comment
Flow onset delay	Δ	1.15 s	metabolism leads flow
Metabolic time constant	$\tau_{m}$	2.99 s	CMRO_2_ rise
Resting mean transit time	$\tau_{0}$	1.70 s	4.55 s in Table 1
Venous stiffness index	β	1.07	0.00 in Table 1
Implied Grubb exponent	α	0.326	0.500 in Table 1
Deflation viscosity	$\tau_{\text{de}}$	10.03 s	late undershoot
Inflation viscosity	$\tau_{\text{in}}$	0.47 s	—
Vasoactive signal decay	$\tau_{z}$	1.12 s	flow pathway
Autoregulatory feedback	$\tau_{f}$	$4.11\;{\text{s}}^{2}$	flow pathway
Metabolic gain	$a_{m}$	0.54 s	drive into equation (36)
Coupling ratio (steady state)	n	3.80	ratio of areas, ∫δf/∫δm
Coupling ratio (peaks)	$n_{\text{peak}}$	5.97	maxδf/maxδm
Fit quality	$R^{2}$	0.9992	vs canonical plus 12% dip

**Table 5.** Dependence of the initial dip on the flow delay

Flow delay Δ (s)	Dip/peak	Nadir latency (s)	Peak latency (s)
0.0	0.000	no dip	—
0.4	−0.022	0.50	4.08
0.8	−0.071	0.94	4.54
1.0	−0.100	1.16	4.78
1.2	−0.129	1.36	5.00
1.6	−0.188	1.76	5.46
2.0	−0.242	2.14	5.90
3.0	−0.340	3.08	6.96

## Data Availability

All the data needed to evaluate the conclusions of the paper are presented in the paper and/or the Supplementary Materials. All analysis code, the parameter files it writes and the raw numerical output are provided as data file S1.

## References

[1] Buxton R. B., Wong E. C., Frank L. R., Dynamics of blood flow and oxygenation changes during brain activation: The balloon model. Magn. Reson. Med. 39, 855–864 (1998). 10.1002/mrm.1910390602 9621908

[2] Ogawa S., Lee T. M., Kay A. R., Tank D. W., Brain magnetic resonance imaging with contrast dependent on blood oxygenation. Proc. Natl. Acad. Sci. U.S.A. 87, 9868–9872 (1990). 10.1073/pnas.87.24.9868 PMC55275 2124706

[3] Biswal B., Yetkin F. Z., Haughton V. M., Hyde J. S., Functional connectivity in the motor cortex of resting human brain using echo-planar MRI. Magn. Reson. Med. 34, 537–541 (1995). 10.1002/mrm.1910340409 8524021

[4] Friston K. J., Mechelli A., Turner R., Price C. J., Nonlinear responses in fMRI: The balloon model, Volterra kernels, and other hemodynamics. NeuroImage 12, 466–477 (2000). 10.1006/nimg.2000.0630 10988040

[5] Glover G. H., Deconvolution of impulse response in event-related BOLD fMRI. NeuroImage 9, 416–429 (1999). 10.1006/nimg.1998.0419 10191170

[6] Lindquist M. A., Loh J. M., Atlas L. Y., Wager T. D., Modeling the hemodynamic response function in fMRI: Efficiency, bias and mis-modeling. NeuroImage 45, S187–S198 (2009). 10.1016/j.neuroimage.2008.10.065 PMC3318970 19084070

[7] Handwerker D. A., Ollinger J. M., D’Esposito M., Variation of BOLD hemodynamic responses across subjects and brain regions and their effects on statistical analyses. NeuroImage 21, 1639–1651 (2004). 10.1016/j.neuroimage.2003.11.029 15050587

[8] Robinson P. A., , BOLD responses to stimuli: Dependence on frequency, stimulus form, amplitude, and repetition rate. NeuroImage 31, 585–599 (2006). 10.1016/j.neuroimage.2005.12.026 16466935

[9] Drysdale P. M., Huber J. P., Robinson P. A., Aquino K. M., Spatiotemporal BOLD dynamics from a poroelastic hemodynamic model. J. Theor. Biol. 265, 524–534 (2010). 10.1016/j.jtbi.2010.05.026 20665966

[10] Aquino K. M., Schira M. M., Robinson P. A., Drysdale P. M., Breakspear M., Hemodynamic traveling waves in human visual cortex. PLOS Comput. Biol. 8, e1002435 (2012). 10.1371/journal.pcbi.1002435 PMC3310706 22457612

[11] Aquino K. M., Robinson P. A., Schira M. M., Breakspear M., Deconvolution of neural dynamics from fMRI data using a spatiotemporal hemodynamic response function. NeuroImage 94, 203–215 (2014). 10.1016/j.neuroimage.2014.03.001 24632091

[12] Pang J. C., Robinson P. A., Aquino K. M., Response-mode decomposition of spatiotemporal hemodynamics. J. R. Soc. Interface 13, 20160253 (2016). 10.1098/rsif.2016.0253 PMC4892270 27170653

[13] Logothetis N. K., Pauls J., Augath M., Trinath T., Oeltermann A., Neurophysiological investigation of the basis of the fMRI signal. Nature 412, 150–157 (2001). 10.1038/35084005 11449264

[14] Hillman E. M. C., Coupling mechanism and significance of the BOLD signal: A status report. Annu. Rev. Neurosci. 37, 161–181 (2014). 10.1146/annurev-neuro-071013-014111 PMC4147398 25032494

[15] Ding Z., , Detection of synchronous brain activity in white matter tracts at rest and under functional loading. Proc. Natl. Acad. Sci. U.S.A. 115, 595–600 (2018). 10.1073/pnas.1711567115 PMC5776967 29282320

[16] Li M., Newton A. T., Anderson A. W., Ding Z., Gore J. C., Characterization of the hemodynamic response function in white matter tracts for event-related fMRI. Nat. Commun. 10, 1140 (2019). 10.1038/s41467-019-09076-2 PMC6408456 30850610

[17] Gore J. C., , Functional MRI and resting state connectivity in white matter — a mini-review. Magn. Reson. Imaging. 63, 1–11 (2019). 10.1016/j.mri.2019.07.017 PMC6861686 31376477

[18] Davis T. L., Kwong K. K., Weisskoff R. M., Rosen B. R., Calibrated functional MRI: Mapping the dynamics of oxidative metabolism. Proc. Natl. Acad. Sci. U.S.A. 95, 1834–1839 (1998). 10.1073/pnas.95.4.1834 PMC19199 9465103

[19] Hoge R. D., , Linear coupling between cerebral blood flow and oxygen consumption in activated human cortex. Proc. Natl. Acad. Sci. U.S.A. 96, 9403–9408 (1999). 10.1073/pnas.96.16.9403 PMC17795 10430955

[20] Blockley N. P., Griffeth V. E. M., Simon A. B., Buxton R. B., A review of calibrated blood oxygenation level-dependent (BOLD) methods for the measurement of task-induced changes in brain oxygen metabolism. NMR Biomed 26, 987–1003 (2013). 10.1002/nbm.2847 PMC3639302 22945365

[21] Obata T., , Discrepancies between BOLD and flow dynamics in primary and supplementary motor areas: Application of the balloon model to the interpretation of BOLD transients. NeuroImage 21, 144–153 (2004). 10.1016/j.neuroimage.2003.08.040 14741651

[22] Grubb R. L., Raichle M. E., Eichling J. O., Ter-Pogossian M. M., The effects of changes in PaCO_2_ on cerebral blood volume, blood flow, and vascular mean transit time. Stroke 5, 630–639 (1974). 10.1161/01.str.5.5.630 4472361

[23] Buxton R. B., Uludağ K., Dubowitz D. J., Liu T. T., Modeling the hemodynamic response to brain activation. NeuroImage 23, S220–S233 (2004). 10.1016/j.neuroimage.2004.07.013 15501093

[24] Stephan K. E., Weiskopf N., Drysdale P. M., Robinson P. A., Friston K. J., Comparing hemodynamic models with DCM. NeuroImage 38, 387–401 (2007). 10.1016/j.neuroimage.2007.07.040 PMC2636182 17884583

[25] Havlicek M., , Physiologically informed dynamic causal modeling of fMRI data. NeuroImage 122, 355–372 (2015). 10.1016/j.neuroimage.2015.07.078 26254113

[26] Ogawa S., , Functional brain mapping by blood oxygenation level-dependent contrast magnetic resonance imaging: A comparison of signal characteristics with a biophysical model. Biophys. J 64, 803–812 (1993). 10.1016/S0006-3495(93)81441-3 PMC1262394 8386018

[27] Yablonskiy D. A., Haacke E. M., Theory of NMR signal behavior in magnetically inhomogeneous tissues: The static dephasing regime. Magn. Reson. Med. 32, 749–763 (1994). 10.1002/mrm.1910320610 7869897

[28] Boxerman J. L., , The intravascular contribution to fMRI signal change: Monte Carlo modeling and diffusion-weighted studies in vivo. Magn. Reson. Med. 34, 4–10 (1995). 10.1002/mrm.1910340103 7674897

[29] Uludağ K., Müller-Bierl B., Uğurbil K., An integrative model for neuronal activity-induced signal changes for gradient and spin echo functional imaging. NeuroImage 48, 150–165 (2009). 10.1016/j.neuroimage.2009.05.051 19481163

[30] Chen J. J., Pike G. B., BOLD-specific cerebral blood volume and blood flow changes during neuronal activation in humans. NMR Biomed 22, 1054–1062 (2009). 10.1002/nbm.1411 19598180

[31] van Zijl P. C. M., Hua J., Lu H., The BOLD poststimulus undershoot, one of the most debated issues in fMRI. NeuroImage 62, 1092–1102 (2012). 10.1016/j.neuroimage.2012.01.029 PMC3356682 22248572

[32] Hua J., Stevens R. D., Huang A. J., Pekar J. J., van Zijl P. C. M., Physiological origin for the BOLD poststimulus undershoot in human brain: Vascular compliance versus oxygen metabolism. J Cereb. Blood. Flow. Metab. 31, 1599–1611 (2011). 10.1038/jcbfm.2011.35 PMC3137471 21468090

[33] Mandeville J. B., , Evidence of a cerebrovascular postarteriole windkessel with delayed compliance. J Cereb. Blood. Flow. Metab. 19, 679–689 (1999). 10.1097/00004647-199906000-00012 10366199

[34] Kong Y., , A model of the dynamic relationship between blood flow and volume changes during brain activation. J Cereb. Blood. Flow. Metab. 24, 1382–1392 (2004). 10.1097/01.WCB.0000141500.74439.53 15625412

[35] Zheng Y., Mayhew J., A time-invariant visco-elastic windkessel model relating blood flow and blood volume. NeuroImage 47, 1371–1380 (2009). 10.1016/j.neuroimage.2009.04.022 PMC2719955 19371789

[36] Boas D. A., Jones S. R., Devor A., Huppert T. J., Dale A. M., A vascular anatomical network model of the spatiotemporal response to brain activation. NeuroImage 40, 1116–1129 (2008). 10.1016/j.neuroimage.2007.12.061 PMC2577617 18289880

[37] Malonek D., Grinvald A., Interactions between electrical activity and cortical microcirculation revealed by imaging spectroscopy: Implications for functional brain mapping. Science 272, 551–554 (1996). 10.1126/science.272.5261.551 8614805

[38] Buxton R. B., The elusive initial dip. NeuroImage 13, 953–958 (2001). 10.1006/nimg.2001.0814 11352601

[39] Uludağ K., To dip or not to dip: Reconciling optical imaging and fMRI data. Proc. Natl. Acad. Sci. U.S.A. 107, E23 (2010). 10.1073/pnas.0914194107 PMC2823892 20142469

[40] Hu X., Yacoub E., The story of the initial dip in fMRI. NeuroImage 62, 1103–1108 (2012). 10.1016/j.neuroimage.2012.03.005 PMC3389272 22426348

[41] Schilling K. G., , Anomalous and heterogeneous characteristics of the BOLD hemodynamic response function in white matter. Cereb. Cortex Commun. 3, tgac035 (2022). 10.1093/texcom/tgac035 PMC9519945 36196360

[42] Simon A. B., Buxton R. B., Understanding the dynamic relationship between cerebral blood flow and the BOLD signal: Implications for quantitative functional MRI. NeuroImage 116, 158–167 (2015). 10.1016/j.neuroimage.2015.03.080 PMC4468003 25862267

[43] Tang L., Avison M. J., Gore J. C., Nonlinear blood oxygen level-dependent responses for transient activations and deactivations in V1—Insights into the hemodynamic response function with the balloon model. Magn. Reson. Imaging 27, 449–459 (2009). 10.1016/j.mri.2008.07.017 PMC2749038 18805666

[44] Griffeth V. E. M., Buxton R. B., A theoretical framework for estimating cerebral oxygen metabolism changes using the calibrated-BOLD method. NeuroImage 58, 198–212 (2011). 10.1016/j.neuroimage.2011.05.077 PMC3187858 21669292

[45] Wang T., Wilkes D. M., Li M., Wu X., Gore J. C., Ding Z., Hemodynamic response function in brain white matter in a resting state. Cereb. Cortex Commun. 1, tgaa056 (2020). 10.1093/texcom/tgaa056 PMC7552822 33073237

[46] Kubíková T., Kochová P., Tomášek P., Witter K., Tonar Z., Numerical and length densities of microvessels in the human brain: Correlation with preferential orientation of microvessels in the cerebral cortex, subcortical gray matter and white matter, pons and cerebellum. J. Chem. Neuroanat. 88, 22–32 (2018). 10.1016/j.jchemneu.2017.11.005 29113946

[47] Harris J. J., Attwell D., The energetics of CNS white matter. J. Neurosci. 32, 356–371 (2012). 10.1523/JNEUROSCI.3430-11.2012 PMC3272449 22219296

[48] Gao Y., Xu L., Schilling K. G., , Myelination selectively modulates BOLD signal in white matter. Res Sq [Preprint]. rs.3.rs-6597153. doi: 10.21203/rs.3.rs-6597153/v1 (2025)

[49] Blockley N. P., Francis S. T., Gowland P. A., Perturbation of the BOLD response by a contrast agent and interpretation through a modified balloon model. NeuroImage 48, 84–93 (2009). 10.1016/j.neuroimage.2009.06.038 19559799

[50] Mullinger K. J., Mayhew S. D., Bagshaw A. P., Bowtell R., Francis S. T., Poststimulus undershoots in cerebral blood flow and BOLD fMRI responses are modulated by poststimulus neuronal activity. Proc. Natl. Acad. Sci. U.S.A. 110, 13636–13641 (2013). 10.1073/pnas.1221287110 PMC3746922 23898206

[51] Kim T., Kim S.-G., Temporal dynamics and spatial specificity of arterial and venous blood volume changes during visual stimulation: Implication for BOLD quantification. J. Cereb. Blood Flow Metab. 31, 1211–1222 (2011). 10.1038/jcbfm.2010.226 PMC3099637 21179068

[52] Huber L., , Validating layer-specific VASO across species. NeuroImage 237, 118195 (2021). 10.1016/j.neuroimage.2021.118195 34038769

[53] Zheng Y., Johnston D., Berwick J., Chen D., Billings S., Mayhew J., A three-compartment model of the hemodynamic response and oxygen delivery to brain. NeuroImage 28, 925–939 (2005). 10.1016/j.neuroimage.2005.06.042 16061400

[54] Huppert T. J., Allen M. S., Benav H., Jones P. B., Boas D. A., A multicompartment vascular model for inferring baseline and functional changes in cerebral oxygen metabolism and arterial dilation. J. Cereb. Blood Flow Metab. 27, 1262–1279 (2007). 10.1038/sj.jcbfm.9600435 PMC2586902 17200678

